# Supplementary material for: Options for reforming agricultural subsidies from health, climate, and economic perspectives
Source: Nat Commun. 2022 Jan 10;13:82. doi: 10.1038/s41467-021-27645-2 (PMC8748512; doi:10.1038/s41467-021-27645-2)
Supplement: Supplementary file 1 — Supplementary Information [file 41467_2021_27645_MOESM1_ESM.pdf]

Supplementary Information for

Options for reforming agricultural subsidies from health, climate, and economic perspectives

M. Springmann, F. Freund

Correspondence to: marco.springmann@ndph.ox.ac.uk or florian.freund@thuenen.de

Contents

Supplementary Methods 2
Supplementary Table 1. 3
Supplementary Table 2. 4
Supplementary Table 3. 5
Supplementary Table 4. 9
Supplementary Table 5. 9
Supplementary Table 6. 11
Supplementary Table 7. 14
Supplementary Table 8. 15
Supplementary Display Items 16
Supplementary Table 9. 16
Supplementary Table 10. 17
Supplementary Figure 1. 18
Supplementary Table 11. 19
Supplementary Table 12. 20
Supplementary Figure 2. 21
Supplementary Figure 3. 22
Supplementary Figure 4. 24
Supplementary Figure 5. 25
Supplementary Figure 6. 27
Supplementary Figure 7. 28
Supplementary Figure 8. 30
Supplementary Figure 9. 31
Supplementary Figure 10. 33
Supplementary Table 13. 34
Supplementary Table 14. 34
Supplementary Table 15. 35
Supplementary Table 16. 35
Supplementary Table 17. 36

Supplementary Methods

Agriculture-economic analysis

We used the Modular Applied General Equilibrium Tool (MAGNET) for the agriculture-economic analysis. MAGNET is a multi-regional, applied computable general equilibrium (CGE) model based on microeconomic theory [1]. The model is solved by finding a price vector that simultaneously clears all factor, goods, and service markets. Different regions in the model are linked via trade flows subject to border policies as well as transportation costs. International trade is governed by the Armington assumption which allows for modelling intra-industrial trade flows in line with observed trade data. The model comprises information on agricultural, industrial and service sectors and is able to capture the interactions between these sectors.

Whilst MAGNET is a model for the whole economy, it has a clear focus on agriculture. It expands the classical economic framework of the Global Trade Analysis Project (GTAP) [2] with respect to land use and land supply, the production of feed and energy crops, and the modelling of agricultural policies. It also features a segmentation of factor markets between agricultural and non-agricultural factors to account for the observable gap between remuneration in those sectors [3].

Household demand is modelled with a non-homothetic Constant Differences in Elasticities (CDE) demand function. This specific form – in contrast to homothetic functions – allows for more realistic non-constant marginal budget shares. Estimates of real (PPP-corrected) GDP per capita were used to account for the observed effects that income elasticities of many food items tend to shrink as income growth [4]. The model includes compensated own-price elasticities, as well as income elasticities. We used income elasticities that, for food commodities, were in line with estimates of the Food and Agriculture Organization of the United Nations (FAO), and we updated the compensated own-price elasticities to the most recent estimates [5] (Supplementary Table 1).

The supply side of the economy is modelled by nested Constant Elasticities of Substitution (CES) functions. Goods and services are produced with a combination of primary production factors (land, labour, capital and natural resources) as well as with domestic and imported intermediates. The production structure therefore resembles the information of input-output tables. The nesting structure allows for different levels of substitutability between different primary production factors and between different intermediates at each nesting level.

In contrast to the basic GTAP model, MAGNET provides a more flexible production structure that also allows for substitutability between specific primary factors and specific intermediates. In an agricultural context this allows, e.g., to model the substitutability between land and fertilizer inputs. Substitution takes place when relative prices change and the degree of substitutability at each stage of the production structure depends on the

respective substitution elasticities. A nested land-use structure accounts for the differences in substitutability of the various types of land use [6,7]. For example, the ease of transforming land used for pasture into land used for the production of sugar crops is different from transforming land used for wheat cultivation into land used for the production of oilseeds.

The model aggregation includes all regions for which information on domestic support payments is available in the GTAP database [8], which corresponds to the information made available by the OECD for specific regions/countries. The remaining countries are grouped into appropriate aggregates (Supplementary Table 2). The sectoral aggregation includes 34 different products (Supplementary Table 3), with agricultural products represented as explicitly as possible given the data provided by GTAP. The 26 agricultural sectors include primary agriculture such as animal husbandry, wheat production and raw milk, processing sectors such as meat production and dairy, as well as sectors for bio energy (bio diesel and bio ethanol). We aggregated the remaining manufacturing and service sectors into one aggregate each, in line with the agricultural focus of our analysis.

MAGNET features a baseline projection that accounts for changes in key economic variables up to the year 2030. Projections of changes in biophysical yields of crops and pastures that account for changes in climate and land use were adapted from the IMAGE integrated-assessment model, and projections of changes in real GDP and population were adopted from the shared socio-economic development pathways developed by the modelling community [9]. For the main analysis, we used a middle-of-the-road development pathway (SSP2), and we considered a more optimistic development pathway (greater economic growth and less population growth, SSP1) and a more pessimistic development pathway (less economic growth, greater population growth, SSP3) in sensitivity analyses.

Supplementary Table 1.

Compensated demand elasticities for selected commodities by region

Commodity	Region grouped by income		
	Low Income	Middle Income	High Income
Wheat	-0.61	-0.55	-0.43
Rice	-0.61	-0.55	-0.43
Other grains	-0.61	-0.55	-0.43
Horticultural products	-0.72	-0.65	-0.53
Vegetable oil	-0.60	-0.54	-0.42
Sugar	-0.74	-0.68	-0.56
Beef	-0.78	-0.72	-0.60
Other meat	-0.78	-0.72	-0.60
Dairy products	-0.78	-0.72	-0.60
Other animal products	-0.54	-0.48	-0.36

Supplementary Table 2.

Regional aggregation. Regional groups include OECD countries with agricultural subsidies (OECD), non-OECD countries with agricultural subsidies (non-OECD), and countries without agricultural subsidies (wo-SUB).

Grouping	Region	Countries
OECD	EU-28	Austria, Belgium, Bulgaria, Croatia, Czech Republic, Cyprus, Denmark, Estonia, France, Finland, Germany, Greece, Hungary, Italy, Latvia, Lithuania, Luxemburg, Malta, Netherlands, Poland, Portugal, Romania, Spain, Slovakia, Slovenia, Sweden, United Kingdom
	USA	USA
	Japan	Japan
	Mexico	Mexico
	Turkey	Turkey
	Switzerland	Switzerland
	Canada	Canada
	South Korea	South Korea
	Norway	Norway
	Australia	Australia
	Chile	Chile
	Israel	Israel
	Rest of EFTA	Iceland, Liechtenstein
	New Zealand	New Zealand
non-OECD	China	China
	India	India
	Russia	Russia
	Brazil	Brazil
	Indonesia	Indonesia
	Kazachstan	Kazachstan
	Colombia	Colombia
	Argentina	Argentina
	Viet Nam	Viet Nam
	Philippines	Philippines
	South Africa	South Africa
	Ukraine	Ukraine
wo-SUB	Rest of Central Asia	Armenia, Azerbaijan, Georgia, Kyrgyztan, Mongolia, Tajikistan, Turkmenistan, Uzbekistan
	Middle East and North Africa (MENA)	United Arab Emirates, Bahrain, Egypt, Iran, Jordhan, Kuwait, Morocco, Oman, Qatar, Saudi Arabia, Tunisia, Algeria, Libya, Western Sahara, Afghanistan, Bhutan, Maldives
	Rest of Asia	Bangladesh, Brunei Darassalam, Cambodia, Lao, Sri Lanka, Malaysia, Nepal, Pakistan, Singapore, Thailand, Democratic People’s Republic of Korea, Macao, Myanmar, Timor-Leste,
	Rest of Latin America and Caribbean	Bolivia, Colombia, Costa Rica, Dominican Republic, Ecuador, Guatemala, Honduras, Jamaica, Nicaragua, Panama, Peru, Puerto Rico, Paraguay, El Salvador, Trinidad and Tobago, Uruguay, Venezuela, Falkland Islands, French Guiana, Guyana, Suriname, Belize, Anguilla, Antigua and Barbados, Aruba, Bahamas, Barbados, British Virgin Islands, Cayman Islands, Cuba, Dominica, Grenada, Haiti, Montserrat, Netherlands Antilles, Saint Kitts and Nevis, Saint Lucia, Saint Vincent and Grenadines, Turks and Caicos Islands, US Virgin Islands
	Rest of Africa	Benin, Burkina Faso, Botswana, Cote d’Ivoire, Cameroon, Ethiopia, Ghana, Guinea, Kenya, Madagascar, Mozambique, Mauritius, Malawi, Namibia, Nigeria, Rwanda, Senegal, Togo, Tanzania, Uganda, Zambia, Zimbabwe, Cape Verde, Gambia, Guinea-Bissau, Liberia, Mali, Mauretania, Niger, Saint Helena, Sierra Leone, Central African Republic, Chad, Congo, Equatorial Guinea, Gabon, Sao Tome and Principe, Angola, Democratic Republic of the Congo, Burundi, Comoros, Djibouti, Eritrea, Mayotte, Seychelles, Somalia, Sudan, Lesotho, Swaziland
	Rest of World	Other Countries

Supplementary Table 3.

Sectoral aggregation

MAGNET aggregate	GTAP sectors	Description
wht	wht	Wheat
gro	gro	Other Cereals and Grains
v_f	v_f	Vegetables, fruits, and other horticultural products
osd	osd	Oil Seeds
c_b	c_b	Sugar Cane and Sugar Beet
pcr	pdr, pcr	Rice
pfb	pfb	Plant based Fibers
oilcake	-	Oilcake
ddgs	-	DDGS
feed	-	Processed Animal Feed
ocr	ocr	Other Crops
ctl	ctl	Cattle, Sheep, Goats, Horses
oap	oap	Pig, Poultry, Eggs
wol	wol	Wool
rmk	rmk	Raw Milk
cmt	cmt	Meat: Cattle, Sheep, Goats, Horses
omt	omt	Meat: Pig and Poultry
vol	vol	Vegetable Oils and Fats
mil	mil	Dairy Products
sgr	sgr	Sugar
ofd	ofd	Food Products
b_t	b_t	Beverages and Tobaccos
fsh	fsh	Forestry
frs	frs	Fishing
biod	-	Biodiesel
biog	-	Biogas
coa	coa	Coal
oil	oil	Crude Oil
gas	gas	Gas
p_c	p_c	Petroleum, Coal Products
ely	ely	Electricity
crp	crp	Chemical Industry
MNFC	ele, fmp, i_s, lea, lum, mvh, nfm, nmm, ome, omf, omn, otn, ppp, tex, wap,	Manufacturing
SEVCS	atp, cmn, cns, dwe, gdt, isr, obs, ofi, osg, otp, ros, trd, wtp, wtr	Services

Note: Sectors with "-" are not part of the GTAP data base and are MAGNET specific sectors. For a detailed sector description see https://www.gtap.agecon.purdue.edu/databases/contribute/detailedsector.asp

Representation of agriculture subsidies

For the study, we relied on subsidy data provided by the OECD for the year 2017. The data has been standardised and processed for use in CGE-based policy analyses in line with GTAP specifications, including a focus on budgetary transfers and mapping of payment categories.

Subsidies in the GTAP dataset are based on information provided by the OECD in their database on Producer Support Estimates (PSE). The PSE data are composed of estimates of Market Price Support (MPS) and budgetary transfers. The MPS estimates consist of policies that alter the national prices of agricultural products and are mostly comprised of border policies such as tariffs. To avoid double counting with tariffs already included in the database, these measures are not included in the GTAP support dataset. The scenario analysis therefore focuses on changes in budgetary transfers in particular.

Budgetary transfers in the PSE dataset are differentiated by different categories. These include (i) payments based on output; (ii) payments based on inputs; (iii) payments based on current or non-current (e.g. historical) area/animal number/returns/investments distinguished whether production is required or not; (iv) payments on non-commodity criteria; and (v) miscellaneous payments. To be consistent with the GTAP database, the categories have been converted into the standard GTAP categories [7] of (i) output payments; (ii) intermediate input payments; (iii) land-based payments; (iv) capital-based payments; and (v) labour-based payments.

PSE estimates also provide information on the degree of decoupling of payments, which are preserved in the converted dataset. The degree of coupling/decoupling is a measure of how far payments are tied to the production of a specific agricultural good. With respect to coupling, the subsidies in the PSE database are grouped into the several categories, including Single Commodity Transfers (SCT), Group Commodity Transfers (GCT), All Commodity Transfers (ACT), and Other Transfers to Producers (OTP). SCT are tied to a specific commodity, whereas ACT and GCT apply to a broader set of commodities, and OTP are provided without requiring production. In the GTAP database, decoupled payments are introduced by equalizing the payment rates for production factors in all eligible agricultural sectors. Thus, the payments introduce no incentive to switch production factors between the eligible sectors, and the associated change in output is minimal.

In the modelling environment, the general representation of agricultural subsidies (PAY) is then based on the introduction of tax wedges between agents and market prices:

$$\begin{aligned} \mathrm{PAY} &= \mathrm{PS} \cdot \mathrm{QO} - \mathrm{PM} \cdot \mathrm{QO} \\ &= \mathrm{PM} \cdot (1 + t_o) \cdot \mathrm{QO} - \mathrm{PM} \cdot \mathrm{QO} \end{aligned}$$

where QO is output and PS is the price payed by the agent (i.e., the farmer) including taxes/subsidies, and PM is the associated market price. The tax variable t_0 is calibrated such that payments (PAY) based on outputs match the support estimates from the OECD database

that are based on output. Similar formulas apply for the case of input and factor-use subsidies. For example, if payments are provided based on crop area as in category (ii), then land-subsidy rates are calibrated accordingly [7].

When model parameters are shocked in a policy simulation, this causes quantities (e.g., QO) and/or prices (e.g., PM) to change endogenously. This would normally also affect the level of agricultural subsidies, but MAGNET allows to exogenise subsidy payments by endogenously shifting tax rates such that a predetermined subsidy level can be achieved. The procedure follows the following equation:

$$(\mathrm{PM} \cdot \mathrm{QO}) \cdot t = 100 \cdot \mathrm{d\_PAY} - \mathrm{PAY}(\mathrm{qo} + \mathrm{pm}).$$

where lower (upper) case variables indicate percentage changes (level) variables. The 'tax-shifter' t is endogenously determined allowing that exogenously specified changes in the support budget (d_PAY) are reached while correcting for endogenous changes in quantities and market prices (as indicated in the second term on the right-hand side of the equation). Similar equations apply to subsidies on inputs and production factors [1].

In our policy simulations, we changed overall support payments (PAY) proportionally and used the remaining subsides for supporting healthier and more environmentally friendly agricultural products. For that purpose, payments were directed to the production of low-emitting and nutrition-sensitive horticultural products, including fruits, vegetables, legumes, and nuts. Technically, this was achieved by increasing output subsidies for that group.

Linkage to production and consumption-based assessments

We linked MAGNET with health and environmental assessments to analyse the full consequences of agricultural subsidy reform. For the environmental assessment, we used MAGNET's estimates of changes in production, applied those to baseline production data, and coupled those to a set of environmental footprints to estimate changes in environmental pollution. For the health assessment, we used MAGNET's estimates of changes in consumption, applied those to baseline consumption data, and used those in a comparative risk assessment of dietary and weight-related risk factors to estimate changes in mortality. These health impacts have economic implications, and we incorporated those by feeding back the impacts that changes in mortality have for population levels and the work force into the MAGNET, which led to adjustments in economic indicators.

Consumption in MAGNET was derived by tracing primary production through all intermediate sectors to the final consumer. For example, fruits and vegetables can be purchased in its raw form directly from the primary sector (v&f), or for example as canned vegetables from the sector of other processed food (ofd), or as part of a prepared meal from the restaurant sector (sevcs). The processed-food and service sectors use intermediate inputs in fixed proportions, which excludes substitution possibilities and therefore limits demand-side responses to changes in output.

For each assessment, we downscaled the results from MAGNET covering 28 regions to the country level (161 countries and regions) based on data of the Food and Agriculture Organization of the United Nations (FAO). By using percentage changes in production and consumption, we preserved the relative distribution across regions. We adopted estimates of food production from the FAO as baseline production data, and we estimated baseline food consumption by adopting estimates of food availability from the FAO's food balance sheets, and adjusting those for the amount of food wasted at the point of consumption [10,11].

Food balance sheets report on the amount of food that is available for human consumption [11]. They reflect the quantities reaching the consumer, but do not include waste from both edible and inedible parts of the food commodity occurring in the household. As such, the amount of food actually consumed may be lower than the quantity shown in the food balance sheet depending on the degree of losses of edible food in the household, e.g. during storage, in preparation and cooking, as plate-waste, or quantities fed to domestic animals and pets, or thrown away.

We followed the waste-accounting methodology developed by the FAO to account for the amount of food wasted at the household level that was not accounted for in food availability estimates [10]. Supplementary Table 4 provides and overview of the parameters used in the calculation, and Supplementary Table 5 provides an overview of the baseline consumption estimates. For each commodity and region, we estimated food consumption by multiplying food availability data with conversion factors (cf) that represent the amount of edible food (e.g. after peeling) and with the percentage of food wasted during consumption (1-wp(cns)).

For roots and tubers, fruits and vegetables, and fish and seafood, we also accounted for the differences in wastage between the proportion that is utilised fresh (pct_{frsh}) and the proportion that utilised in processed form (pct_{prcd}). The equation used for each food commodity and region was:

$$\text{Consumption} = \text{Availability} \cdot \frac{pct_{frsh}}{100} \cdot cf_{frsh} \cdot \left(1 - \frac{wp(cns_{frsh})}{100}\right) + \text{Availability} \cdot \frac{pct_{prcd}}{100} \cdot cf_{prcd} \cdot \left(1 - \frac{wp(cns_{prcd})}{100}\right)$$

Supplementary Table 4.

Percentage of food wasted (wp) during consumption (cns), and percentage of processed utilisation (pct_{prcd}). The percentage of fresh utilisation is calculated as 1-pct_{prcd}. Conversion factors to edible portions of foods are provided below the table.

Food group	Item	Region: Europe	USA, Canada, Oceania	Indus-trialized Asia	Sub-Saharan Africa	North Africa, West and Central Asia	South and Southeast Asia	Latin America
cereals	wp(cns)	25	27	20	1	12	3	10
roots and tuber	pct_{prcd}	73	73	15	50	19	10	80
	wp(cns)	17	30	10	2	6	3	4
	wp(cns_{prcd})	12	12	12	1	3	5	2
oilseeds and pulses	cns	4	4	4	1	2	1	2
fruits and vegetables	pct_{prcd}	60	60	4	1	50	5	50
	wp(cns)	19	28	15	5	12	7	10
	wp(cns_{prcd})	15	10	8	1	1	1	1
milk and dairy	wp(cns)	7	15	5	0.1	2	1	4
eggs	wp(cns)	8	15	5	1	12	2	4
meat	wp(cns)	11	11	8	2	8	4	6
fish and seafood	pct_{prcd}	*40% for low-income countries, and 96% for all others.*						
	wp(cns)	11	33	8	2	4	2	4
	wp(cns_{prcd})	10	10	7	1	2	1	2

Conversion factors: maize, millet, sorghum: 0.69; wheat, rye, other grains: 0.78; rice: 1; roots: 0.74 (0.9 for industrial processing); nuts and seeds: 0.79; oils: 1; vegetables: 0.8 (0.75 for industrial processing); fruits: 0.8 (0.75 for industrial processing); beef: 0.715; lamb: 0.71; pork: 0.68; poultry: 0.71; other meat: 0.7; milk and dairy: 1; fish and seafood: 0.5; other crops: 0.78

Supplementary Table 5.

Food consumption in the year 2030 (in grams per day per person) by region and food group

Food group	Region: World	OECD	non-OECDs	wo-SUB
Wheat	124	137	126	114
Rice	129	27	178	112
Other grains	59	43	36	102
Fruits & veg	587	534	639	539
Vegetable oil	40	58	37	34
Oilseeds	4	3	5	2
Sugar	48	72	43	43
Beef & lamb	26	46	19	25
Pork & poultry	53	102	55	24
Dairy products	233	480	175	182
Other animal products	25	34	31	11
Other food	108	185	93	86

Environmental analysis

In our environmental analysis, we focused on changes in GHG emissions, because those most directly relate to dietary changes and are relatively less modifiable by changes in farm-level management [12]. We estimated the emissions impacts of agricultural subsidy reform by pairing the changes in agricultural production with a global dataset of country and crop-specific emissions footprints [12].

In our analysis, agricultural GHG emissions include methane and nitrous oxide, whilst carbon dioxide emissions are allocated to other sectors (e.g., energy and transport) in line with methodology followed by the International Panel on Climate Change. The data on GHG emissions were adopted from country-specific analyses of GHG emissions from crops [13], and livestock [14]. Non-CO_2 emissions of fish and seafood were calculated based on feed requirements and feed-related emissions of aquaculture [15], and on projections of the ratio between wild-caught and farmed fish production [16,17].

For future years, we incorporated the mitigation potential of bottom-up changes in management practices and technologies by using marginal abatement cost curves [18], and the projected value of the social cost of carbon (SCC) in that year [19]. The mitigation options included changes in irrigation, cropping and fertilization that reduce methane and nitrous oxide emissions for rice and other crops, as well as changes in manure management, feed conversion and feed additives that reduce enteric fermentation in livestock.

Along a middle-of-the-road development pathway (SSP2), these options resulted in a reduction in food-related greenhouse gas emissions of 6% on average, ranging from 3-9% for countries grouped by income (i.e., from high to low-income countries). No improvements in management practices and technologies were included a more pessimistic development pathway (SSP3) to allow for additional sensitivity analyses. Our baseline emissions estimate agrees well with existing ones that follow the same methodology [20,21]. Supplementary Table 6 provides an overview of the emissions footprints used in the analysis.

Supplementary Table 6.

GHG emissions footprints by food group and region (kgCO_2-eq per kg of product).

Food group	Region			
	Global	OECD	non-OECD	wo-SUB
wheat	0.22	0.24	0.21	0.22
rice	1.02	0.99	1.27	0.86
maize	0.18	0.18	0.17	0.20
other grains	0.31	0.38	0.33	0.19
roots	0.07	0.10	0.07	0.05
legumes	0.21	0.19	0.27	0.20
soybeans	0.11	0.14	0.11	0.06
vegetables	0.06	0.05	0.07	0.07
oilcrops	0.71	0.64	0.50	1.05
sugarcrops	0.01	0.03	0.01	0.01
temperate fruits	0.08	0.05	0.09	0.09
tropical fruits	0.15	0.03	0.31	0.07
starchy fruits	0.10	0.02	0.27	0.07
milk	1.10	0.64	1.28	1.55
eggs	1.45	1.34	1.55	1.35
beef	30.62	15.17	35.68	45.86
lamb	30.68	23.79	24.35	38.28
pork	2.70	2.58	2.52	3.95
poultry	1.32	0.96	1.62	1.49
shellfish	0.04	0.01	0.03	0.06
fish (freshwater)	0.11	0.15	0.13	0.08
fish (demersal)	0.01	0.01	0.01	0.02

Health analysis

We estimated the mortality and disease burden attributable to dietary and weight-related risk factors by calculating population impact fractions (PIFs) which represent the proportions of disease cases that would be avoided when the risk exposure was changed from a baseline situation to a counterfactual situation. For calculating PIFs, we used the general formula [22,23]:

$$\mathrm{PIF} = \frac{\int \mathrm{RR}(x)P(x)dx - \int \mathrm{RR}(x)P'(x)dx}{\int \mathrm{RR}(x)P(x)dx}$$

where $\mathrm{RR}(x)$ is the relative risk of disease for risk factor level x, $P(x)$ is the number of people in the population with risk factor level x in the baseline scenario, and $P'(x)$ is the number of people in the population with risk factor level x in the counterfactual scenario. We assumed that changes in relative risks follow a dose-response relationship, and that PIFs combine multiplicatively, i.e. $\mathrm{PIF} = 1 - \prod_i(1 - \mathrm{PIF_i})$ where the *i*'s denote independent risk factors [22,23].

The number of avoided deaths due to the change in risk exposure of risk i, $\Delta\mathrm{deaths_i}$, was calculated by multiplying the associated PIF by disease-specific death rates, DR, and by the number of people alive within a population, *P*:

$$\Delta\mathrm{deaths_i}(r, a, d) = \mathrm{PIF_i}(r, d) \cdot \mathrm{DR}(r, a, d) \cdot P(r, a)$$

where PIFs are differentiated by region *r* and disease/cause of death *d*; the death rates are differentiated by region, age group *a*, and disease; the population groups are differentiated by region and age group; and the change in the number of deaths is differentiated by region, age group and disease.

We used publicly available data sources to parameterize the comparative risk analysis. Mortality and population data were adopted from the Global Burden of Disease project [24]. Baseline data on the weight distribution in each country were adopted from a pooled analysis of population-based measurements undertaken by the NCD Risk Factor Collaboration [25] and projected forward based on the statistical relationship between calorie availability and body weight [26].

The relative risk estimates that relate the risk factors to the disease endpoints were adopted from meta-analyses of prospective cohort studies for dietary weight-related risks [27–34]. In line with the meta-analyses, we included non-linear dose-response relationships for fruits and vegetables, nuts and seeds, and fish, and assumed linear dose-response relationships for the remaining risk factors. As our analysis was primarily focused on mortality from chronic diseases, we focused on adults aged 20 year or older, and we adjusted the relative-risk

estimates for attenuation with age based on a pooled analysis of cohort studies focussed on metabolic risk factors [35], in line with other assessments [36,37]. Supplementary Table 7 provides an overview of the relative-risk parameters used.

The selection of risk-disease associations used in the health analysis was supported by available criteria used to judge the certainty of evidence, such as the Bradford-Hill criteria used by the Nutrition and Chronic Diseases Expert Group (NutriCoDE) [37], the World-Cancer-Research-Fund criteria used by the Global Burden of Disease project [38], as well as NutriGrade (Supplementary Table 8) [39]. The certainty of evidence supporting the associations of dietary risks and disease outcomes as used here were graded as moderate or high with NutriGrade [27,31,32], and/or assessed as probable or convincing by the Nutrition and Chronic Diseases Expert Group [37], and by the World Cancer Research [40]. The certainty of evidence grading in each case relates to the general relationship between a risk factor and a health outcome, and not to a specific relative-risk value.

For the different diet scenarios, we calculated uncertainty intervals associated with changes in mortality based on standard methods of error propagation and the confidence intervals of the relative risk parameters. For the error propagation, we approximated the error distribution of the relative risks by a normal distribution and used that side of deviations from the mean which was largest. This method leads to conservative and potentially larger uncertainty intervals as probabilistic methods, such as Monte Carlo sampling, but it has significant computational advantages, and is justified for the magnitude of errors dealt with here (<50%) (see e.g. IPCC Uncertainty Guidelines).

Supplementary Table 7.

Relative risk parameters (mean and low and high values of 95% confidence intervals) for dietary risks and weight-related risks.

Food group	Endpoint	Unit	RR mean	RR low	RR high	Reference
Red meat	CHD	100 g/d	1.15	1.08	1.23	Bechthold et al (2019)
	Stroke	100 g/d	1.12	1.06	1.17	Bechthold et al (2019)
	Colorectal cancer	100 g/d	1.12	1.06	1.19	Schwingshackl et al (2018)
	Type 2 diabetes	100 g/d	1.17	1.08	1.26	Schwingshackl et al (2017)
Fish	CHD	15 g/d	0.94	0.90	0.98	Zheng et al (2012)
Fruits	CHD	100 g/d	0.95	0.92	0.99	Aune et al (2017)
	Stroke	100 g/d	0.77	0.70	0.84	Aune et al (2017)
	Cancer	100 g/d	0.94	0.91	0.97	Aune et al (2017)
Vegetables	CHD	100 g/d	0.84	0.80	0.88	Aune et al (2017)
	Cancer	100 g/d	0.93	0.91	0.95	Aune et al (2017)
Legumes	CHD	57 g/d	0.86	0.78	0.94	Afshin et al (2014)
Nuts	CHD	28 g/d	0.71	0.63	0.80	Aune et al (2016)
Underweight	CHD	15<BMI<18.5	1.17	1.09	1.24	Global BMI Collab (2016)
	Stroke	15<BMI<18.5	1.37	1.23	1.53	Global BMI Collab (2016)
	Cancer	15<BMI<18.5	1.10	1.05	1.16	Global BMI Collab (2016)
	Respiratory disease	15<BMI<18.5	2.73	2.31	3.23	Global BMI Collab (2016)
Overweight	CHD	25<BMI<30	1.34	1.32	1.35	Global BMI Collab (2016)
	Stroke	25<BMI<30	1.11	1.09	1.14	Global BMI Collab (2016)
	Cancer	25<BMI<30	1.10	1.09	1.12	Global BMI Collab (2016)
	Respiratory disease	25<BMI<30	0.90	0.87	0.94	Global BMI Collab (2016)
	Type 2 diabetes	25<BMI<30	1.88	1.56	2.11	Prosp Studies Collab (2009)
Obesity (grade 1)	CHD	30<BMI<35	2.02	1.91	2.13	Global BMI Collab (2016)
	Stroke	30<BMI<35	1.46	1.39	1.54	Global BMI Collab (2016)
	Cancer	30<BMI<35	1.31	1.28	1.34	Global BMI Collab (2016)
	Respiratory disease	30<BMI<35	1.16	1.08	1.24	Global BMI Collab (2016)
	Type 2 diabetes	30<BMI<35	3.53	2.43	4.45	Prosp Studies Collab (2009)
Obesity (grade 2)	CHD	30<BMI<35	2.81	2.63	3.01	Global BMI Collab (2016)
	Stroke	30<BMI<35	2.11	1.93	2.30	Global BMI Collab (2016)
	Cancer	30<BMI<35	1.57	1.50	1.63	Global BMI Collab (2016)
	Respiratory disease	30<BMI<35	1.79	1.60	1.99	Global BMI Collab (2016)
	Type 2 diabetes	30<BMI<35	6.64	3.80	9.39	Prosp Studies Collab (2009)
Obesity (grade 3)	CHD	30<BMI<35	3.81	3.47	4.17	Global BMI Collab (2016)
	Stroke	30<BMI<35	2.33	2.05	2.65	Global BMI Collab (2016)
	Cancer	30<BMI<35	1.96	1.83	2.09	Global BMI Collab (2016)
	Respiratory disease	30<BMI<35	2.85	2.43	3.34	Global BMI Collab (2016)
	Type 2 diabetes	30<BMI<35	12.49	5.92	19.82	Prosp Studies Collab (2009)

Supplementary Table 8.

Overview of existing ratings on the certainty of evidence for a statistically significant association between a risk factor and a disease endpoint. The ratings include those of the Nutrition and Chronic Diseases Expert Group (NutriCoDE), the World Cancer Research Fund, and NutriGrade.

Food group	Endpoint	Association	Certainty of evidence
Fruits	CHD	reduction	NutriCoDE: probable or convincing;<br>NutriGrade: moderate quality of meta-evidence
	Stroke	reduction	NutriCoDE: probable or convincing<br>NutriGrade: moderate quality of meta-evidence
	Cancer	reduction	WCRF: strong evidence (probable) for some cancers<br>NutriGrade: moderate quality of meta-evidence for colorectal cancer
Vegetables	CHD	reduction	NutriCoDE: probable or convincing<br>NutriGrade: moderate quality of meta-evidence
	Cancer	reduction	WCRF: strong evidence (probable) for non-starchy vegetables and some cancers<br>NutriGrade: moderate quality of meta-evidence for colorectal cancer
Legumes	CHD	reduction	NutriCoDE: probable or convincing<br>NutriGrade: moderate quality of meta-evidence
Nuts and seeds	CHD	reduction	NutriCoDE: probable or convincing<br>NutriGrade: moderate quality of meta-evidence
Fish	CHD	reduction	NutriCoDE: probable or convincing<br>NutriGrade: moderate quality of meta-evidence
Red meat	CHD	increase	NutriGrade: moderate quality of meta-evidence
	Stroke	increase	NutriGrade: moderate quality of meta-evidence
	Cancer	increase	WCRF: strong evidence (probable) for colorectal cancer<br>NutriGrade: moderate quality of meta-evidence for colorectal cancer
	Type-2 diabetes	increase	NutriCoDE: probable or convincing<br>NutriGrade: high quality of meta-evidence

NutriCoDE: Nutrition and Chronic Diseases Expert Group

NutriGrade: Grading of Recommendations Assessment, Development, and Evaluation (GRADE) tailored to nutrition research

WCRF: World Cancer Research Fund

Supplementary Display Items

Supplementary Table 9.

Overview of subsidy payments by type.

Region	Country	Subsidies (USD mill)	Distribution of subsidies by type (%)			
			Single Commodity Transfers	Group Commodity Transfers	All Commodity Transfers	Other Transfers to Producers
World		232,904	8%	29%	31%	31%
OECD		127,628	11%	10%	30%	49%
non-OECD		105,276	5%	57%	32%	6%
OECD	EU	74,171	4%	6%	30%	60%
	USA	28,339	20%	11%	24%	46%
	Japan	8,040	31%	1%	21%	48%
	Mexico	2,898	15%	29%	56%	0%
	Turkey	2,781	48%	35%	17%	0%
	Switzeraland	3,516	9%	27%	32%	31%
	Canada	1,770	29%	24%	46%	1%
	South Korea	2,898	0%	29%	20%	51%
	Norway	1,677	14%	51%	35%	1%
	Australia	779	0%	7%	55%	37%
	Chile	366	0%	5%	95%	0%
	Israel	254	10%	11%	76%	4%
	Iceland	114	97%	0%	3%	0%
	New Zealand	25	0%	100%	0%	0%
non-OECD	China	58,852	2%	56%	32%	10%
	India	35,248	0%	65%	35%	0%
	Russia	3,207	15%	73%	7%	5%
	Brazil	2,263	36%	7%	57%	0%
	Indonesia	3,080	7%	67%	26%	0%
	Kazakhstan	848	24%	69%	7%	1%
	Columbia	462	15%	0%	85%	0%
	Argentina	331	9%	15%	77%	0%
	Vietnam	331	23%	68%	9%	0%
	Philippines	258	51%	35%	12%	2%
	South Africa	205	0%	0%	100%	0%
	Ukraine	191	6%	74%	20%	0%

Supplementary Table 10.

Overview of subsidy payments with attribution to final use.

Region	Country	Subsidies (USD mill)	Distribution of subsidies by food group					
			staples	fruits&veg	oil&sugar	meat	milk	other
World		232,904	52,769	56,736	27,629	48,134	21,949	25,687
OECD		127,628	25,324	23,628	14,211	34,959	18,733	10,773
non-OECD		105,276	27,445	33,108	13,418	13,175	3,216	14,913
OECD	EU	74,171	10,993	13,187	7,756	23,315	13,376	5,544
	USA	28,339	7,740	4,849	4,739	6,201	2,256	2,553
	Japan	8,040	3,272	1,894	687	942	768	478
	Mexico	2,898	604	922	133	915	163	161
	Turkey	2,781	745	603	297	431	91	615
	Switzeraland	3,516	231	497	164	852	1,164	609
	Canada	1,770	424	178	296	369	87	416
	South Korea	2,898	929	1,023	51	644	108	143
	Norway	1,677	172	62	2	765	520	156
	Australia	779	172	130	74	276	66	62
	Chile	366	31	183	10	103	29	10
	Israel	254	11	100	3	90	25	25
	Iceland	114	0	0	0	46	67	1
	New Zealand	25	0	0	0	10	14	1
non-OECD	China	58,852	14,101	23,923	6,545	9,051	636	4,597
	India	35,248	9,909	7,569	5,074	1,927	1,344	9,426
	Russia	3,207	557	649	156	939	898	8
	Brazil	2,263	412	217	772	491	106	264
	Indonesia	3,080	1,794	305	621	127	7	226
	Kazakhstan	848	157	159	78	299	124	31
	Columbia	462	76	94	22	125	54	91
	Argentina	331	49	26	82	82	24	68
	Vietnam	331	207	31	27	15	1	49
	Philippines	258	140	71	20	24	0	3
	South Africa	205	30	55	15	82	13	9
	Ukraine	191	15	8	7	13	8	140

Supplementary Figure 1.

Overview of agricultural support measures in 2017, including major spenders and the distribution by final commodity (total subsidy payments are shown on the right axis and percentage distribution on the left axis).

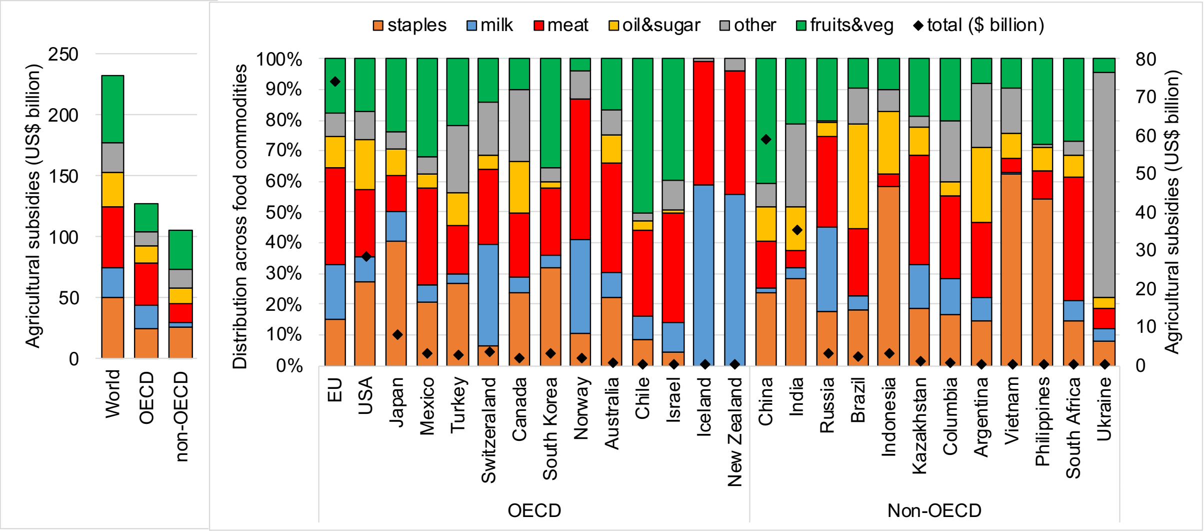

Supplementary Table 11.

Subsidy payments directed towards horticultural commodities in the different scenarios. BMK denotes the baseline (benchmark), and the reform scenarios include a conditioning of 25-100% (S25-S100) of subsidies to food commodities with beneficial health and environmental characteristics, and a combination of conditioning and regional restructuring in which each country provides subsidies in proportion to either its economy (GDP) or population (POP), whilst keeping the global amount of subsidy payments fixed.

Region	Country	Subsidies for horticultural commodities by scenarios (USD million)						
		BMK	S25	S50	S75	S100	GDP	POP
World	Total	56,737	100,779	144,821	188,863	232,905	232,905	232,905
OECD countries	EU	13,187	28,433	43,679	58,925	74,171	41,446	15,645
	USA	4,849	10,722	16,594	22,467	28,339	51,365	10,371
	Japan	1,894	3,431	4,967	6,504	8,040	12,156	3,629
	South Korea	1,023	1,492	1,961	2,430	2,898	3,704	1,490
	Mexico	922	1,416	1,910	2,404	2,898	3,441	4,037
	Turkey	603	1,148	1,692	2,237	2,781	2,709	2,472
	Switzerland	497	1,252	2,007	2,761	3,516	1,586	249
	Chile	184	229	275	320	366	780	567
	Canada	178	576	974	1,372	1,770	4,593	1,182
	Australia	130	292	455	617	779	3,770	819
	Israel	100	139	177	216	254	918	312
	Norway	62	466	869	1,273	1,677	1,239	171
	Iceland	0	29	57	86	114	56	12
	New Zealand	0	6	12	19	25	411	151
Non-OECD countries	China	23,923	32,655	41,387	50,120	58,852	39,613	40,572
	India	7,569	14,489	21,408	28,328	35,248	9,528	42,583
	Russia	649	1,288	1,928	2,567	3,207	6,558	4,142
	Indonesia	305	999	1,692	2,386	3,080	3,842	7,891
	Brazil	217	728	1,240	1,751	2,263	10,288	6,424
	Kazakhstan	159	331	503	675	848	797	532
	Colombia	94	186	278	370	462	1,179	1,623
	Philippines	71	118	165	211	258	950	3,504
	South Africa	55	93	130	168	205	1,369	1,667
	Viet Nam	31	106	181	256	331	790	2,886
	Argentina	26	103	179	255	331	2,748	1,329
	Ukraine	8	54	100	145	191	683	1,262
Non-subsidising countries	MENA	0	0	0	0	0	10,393	13,658
	Rest of Africa	0	0	0	0	0	5,407	33,330
	Rest of South America	0	0	0	0	0	3,799	5,961
	Rest of Asia	0	0	0	0	0	5,311	21,367
	Rest of Central Asia	0	0	0	0	0	701	2,109
	Rest of the World	0	0	0	0	0	776	960

Supplementary Table 12.

Price changes (%) for the different scenario by food commodity and region

Commodity	Region	Scenarios						
		S50	S75	S100	S25	RMV	GDP	POP
wheat	World	2.65	4.04	5.51	1.31	4.15	5.22	4.55
	OECD	4.37	6.71	9.21	2.15	6.63	8.20	7.25
	non-OECD	2.05	3.11	4.23	1.02	3.30	4.19	3.61
rice	World	1.38	2.16	3.00	0.67	2.57	2.87	3.00
	OECD	3.25	5.26	7.57	1.50	6.56	7.98	7.05
	non-OECD	1.11	1.70	2.33	0.55	1.99	2.11	2.40
other grains	World	1.15	1.76	2.39	0.57	2.04	2.29	2.15
	OECD	3.47	5.20	6.96	1.74	5.27	6.66	5.80
	non-OECD	0.93	1.42	1.95	0.46	1.73	1.87	1.79
fruit&veg	World	-4.59	-6.61	-8.58	-2.39	2.24	-10.03	-10.06
	OECD	-9.73	-13.75	-17.58	-5.17	3.45	-18.69	-8.12
	non-OECD	-2.48	-3.63	-4.75	-1.28	1.76	-6.34	-10.88
oil seeds	World	1.29	2.01	2.81	0.63	2.31	2.63	2.45
	OECD	3.11	4.86	6.82	1.51	5.53	6.79	5.98
	non-OECD	1.07	1.66	2.32	0.52	1.92	2.12	2.00
veg oil	World	0.41	0.68	1.02	0.19	1.03	0.97	0.87
	OECD	0.48	0.75	1.05	0.23	0.91	1.04	0.87
	non-OECD	0.40	0.67	1.01	0.18	1.06	0.96	0.87
sugar	World	0.49	0.74	1.00	0.24	0.81	0.88	0.88
	OECD	0.73	1.11	1.49	0.37	1.12	1.41	1.24
	non-OECD	0.40	0.61	0.83	0.20	0.70	0.69	0.75
beef&lamb	World	0.77	1.18	1.61	0.38	1.46	1.66	1.59
	OECD	1.30	1.99	2.71	0.64	2.36	2.70	2.43
	non-OECD	0.29	0.45	0.62	0.14	0.66	0.74	0.82
other aniprod	World	1.06	1.63	2.24	0.52	1.90	2.00	2.17
	OECD	2.23	3.37	4.57	1.10	3.44	4.45	3.85
	non-OECD	0.88	1.36	1.87	0.43	1.66	1.62	1.91
pork&poultry	World	1.16	1.77	2.41	0.58	1.79	2.15	1.98
	OECD	1.78	2.69	3.64	0.88	2.55	3.23	2.77
	non-OECD	0.40	0.62	0.87	0.19	0.84	0.81	1.00
dairy	World	0.75	1.13	1.52	0.38	1.23	1.40	1.29
	OECD	1.11	1.66	2.21	0.56	1.72	2.06	1.85
	non-OECD	0.35	0.54	0.74	0.17	0.67	0.67	0.64
other food	World	-0.03	-0.02	0.02	-0.03	0.72	0.01	0.01
	OECD	0.10	0.18	0.28	0.04	0.63	0.16	0.31
	non-OECD	-0.18	-0.24	-0.27	-0.11	0.83	-0.15	-0.34

Supplementary Figure 2.

Absolute and percentage changes in agricultural production by food group and region in the different reform scenarios.

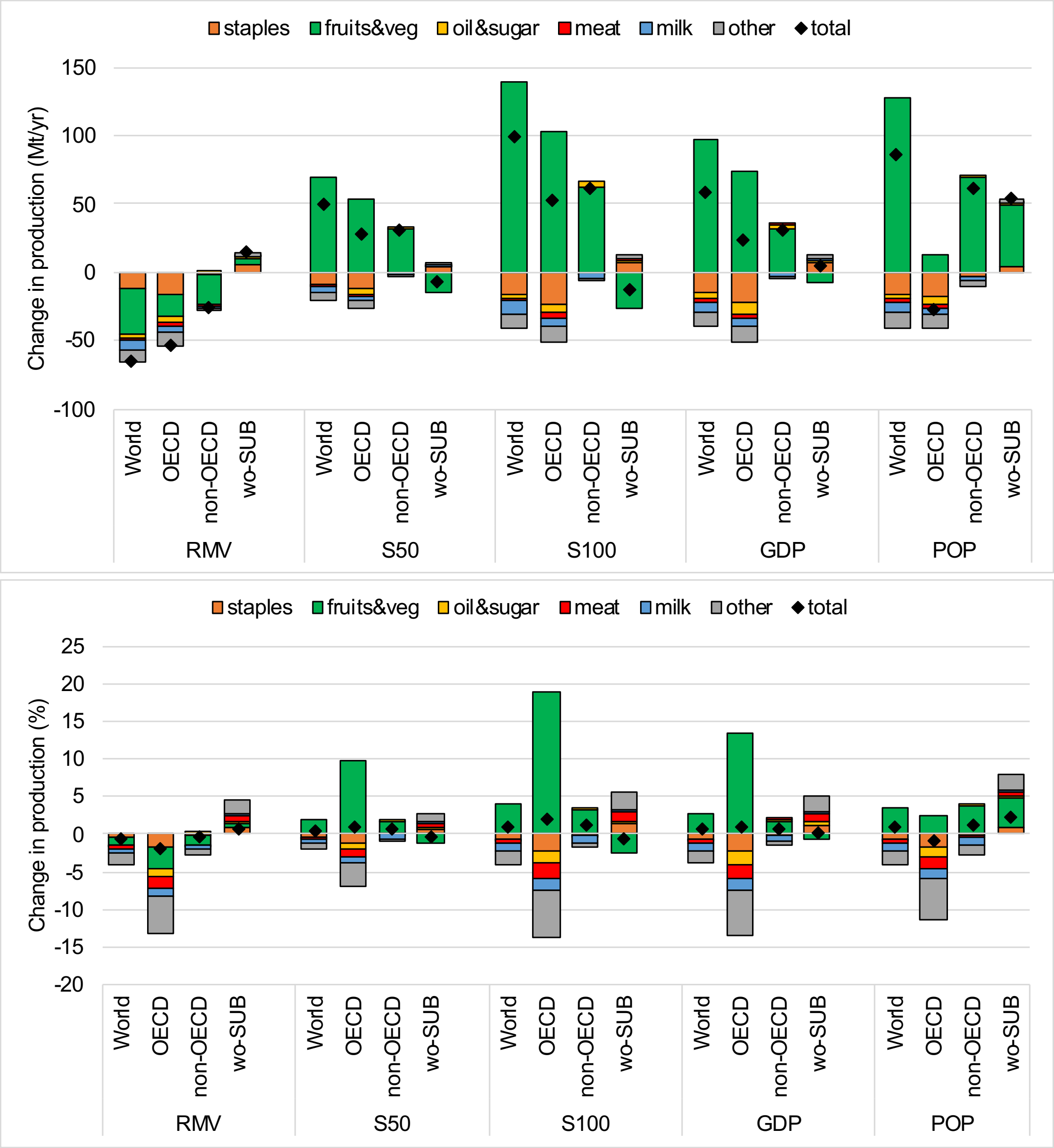

Supplementary Figure 3.

Regional changes in horticultural production (%) by scenario.

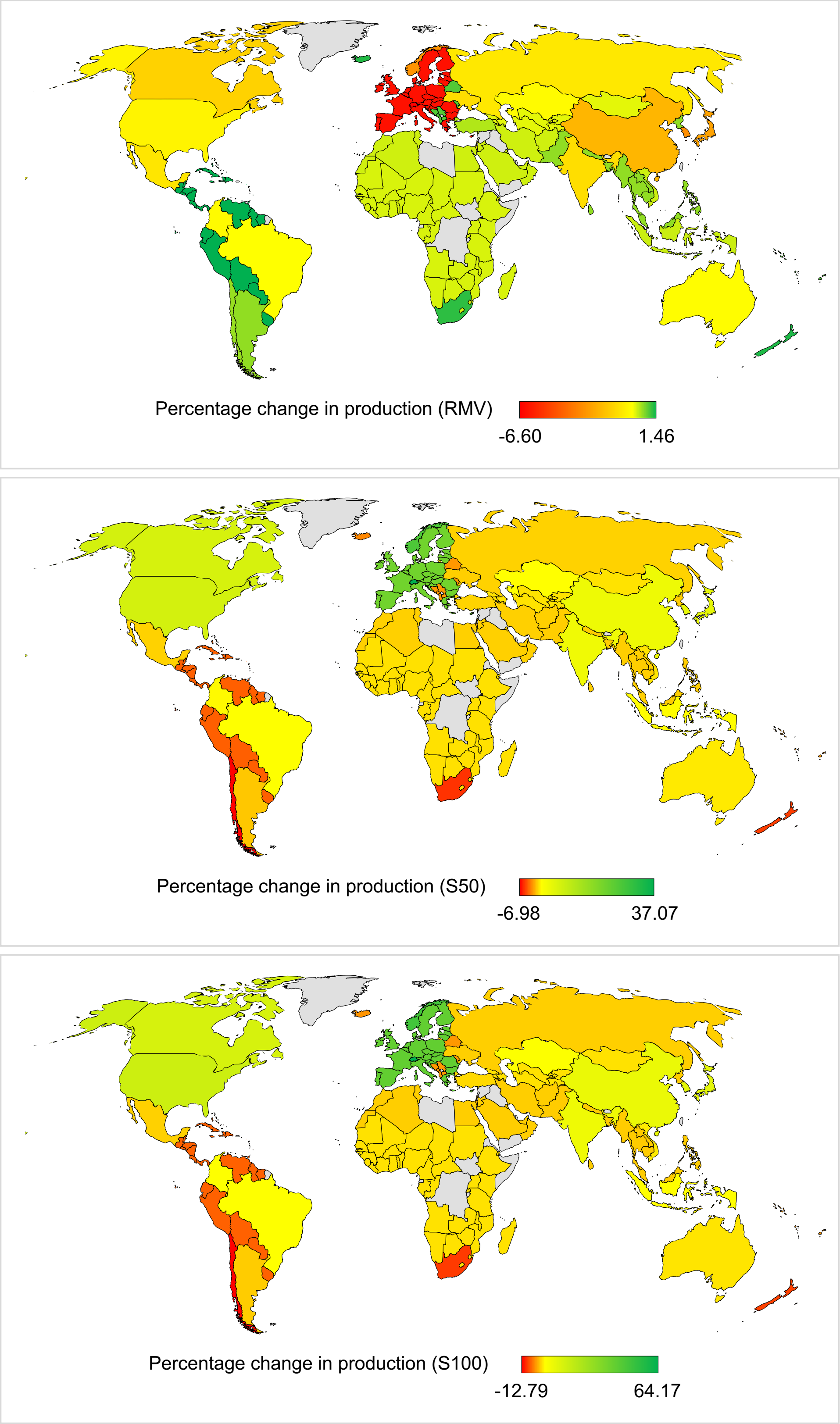

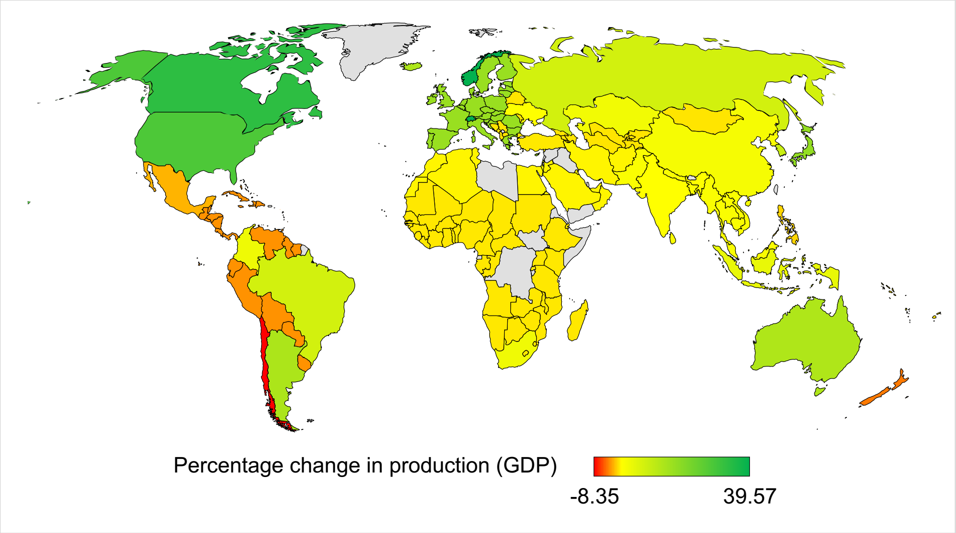
Percentage change in production (GDP)
-8.35
39.57

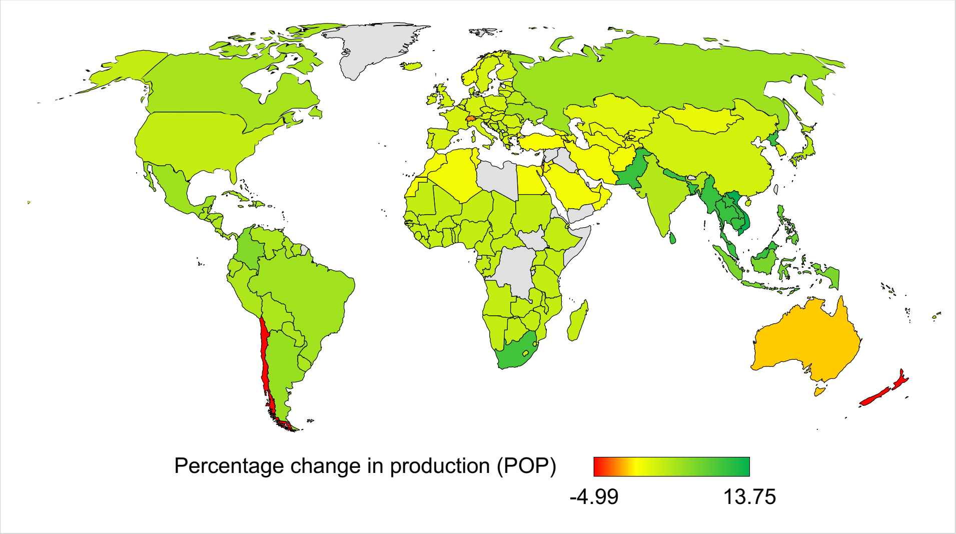
Percentage change in production (POP)
-4.99
13.75

Supplementary Figure 4.

Absolute and percentage changes in food-related GHG emissions by food group and region in the different reform scenarios.

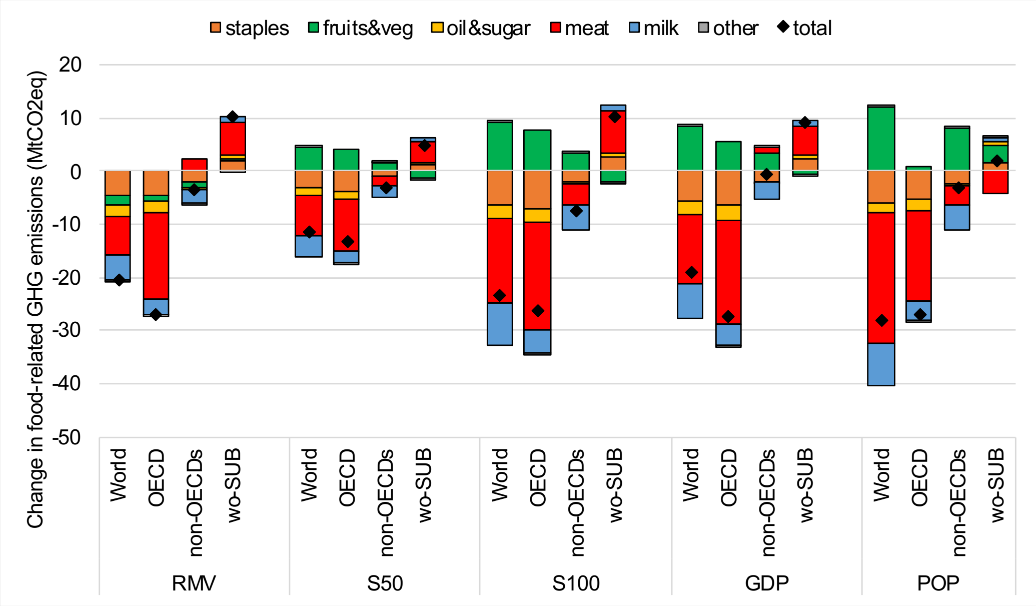

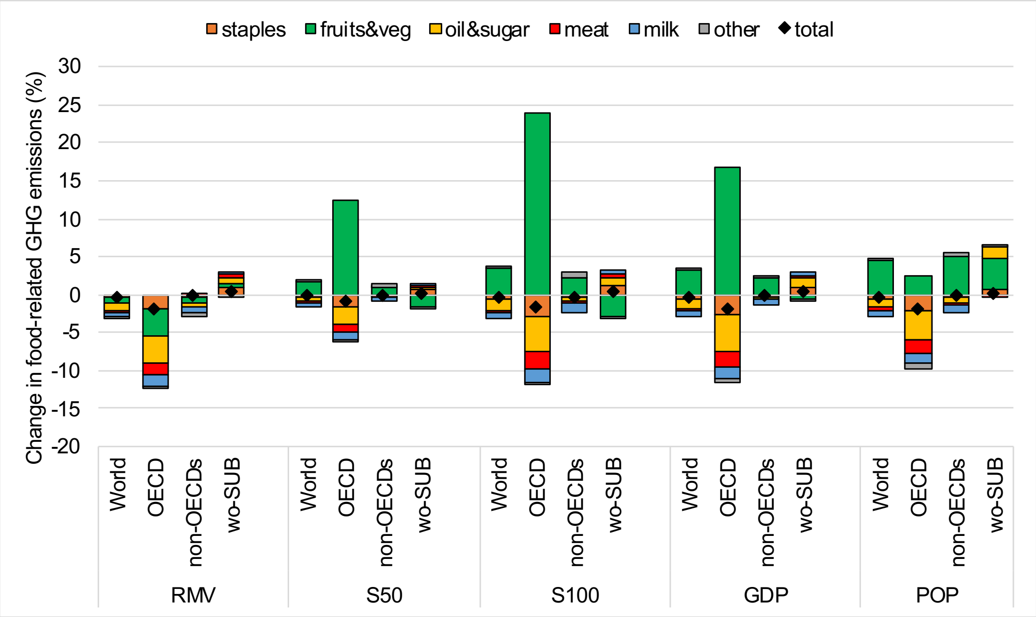

Supplementary Figure 5.

Regional changes in GHG emissions (%) by scenario.

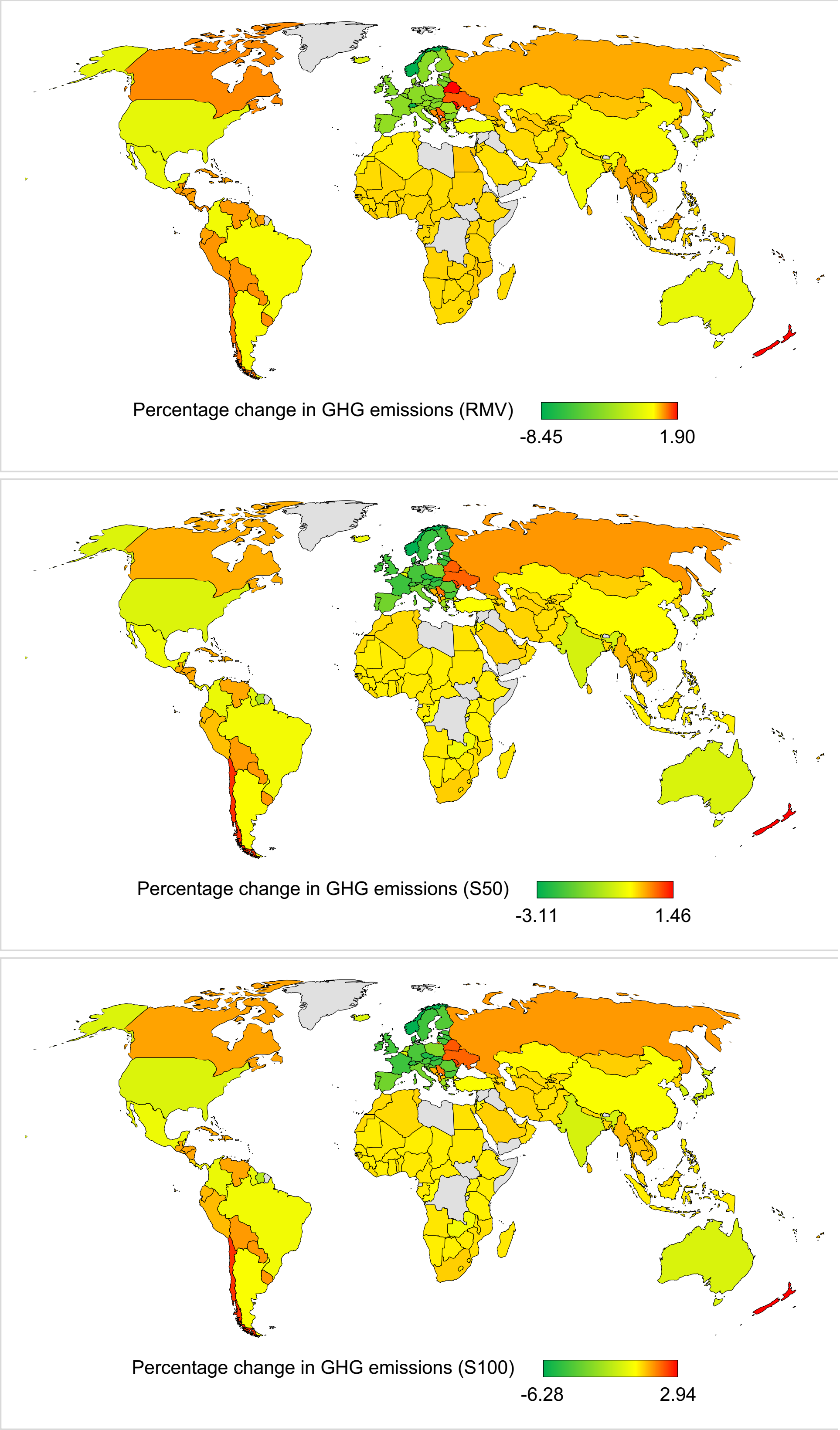

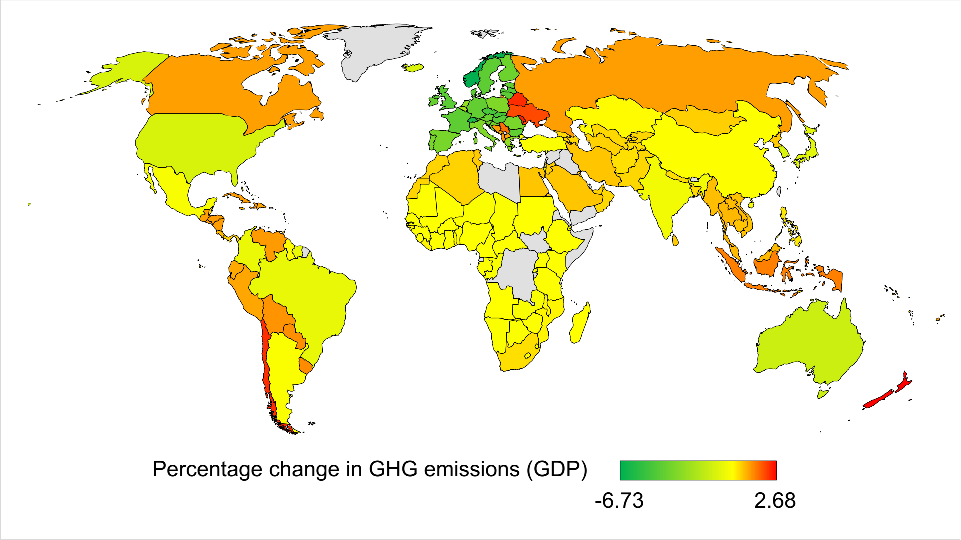
Percentage change in GHG emissions (GDP)
-6.73
2.68

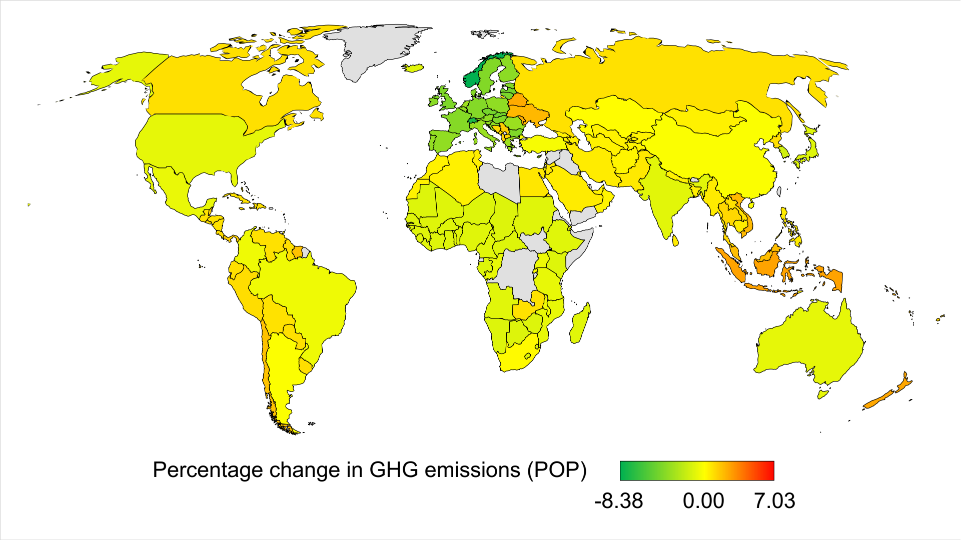
Percentage change in GHG emissions (POP)
-8.38
0.00
7.03

Supplementary Figure 6.

Absolute and percentage changes in food consumption by food group (in g/d and total kcal/d) and region in the different reform scenarios.

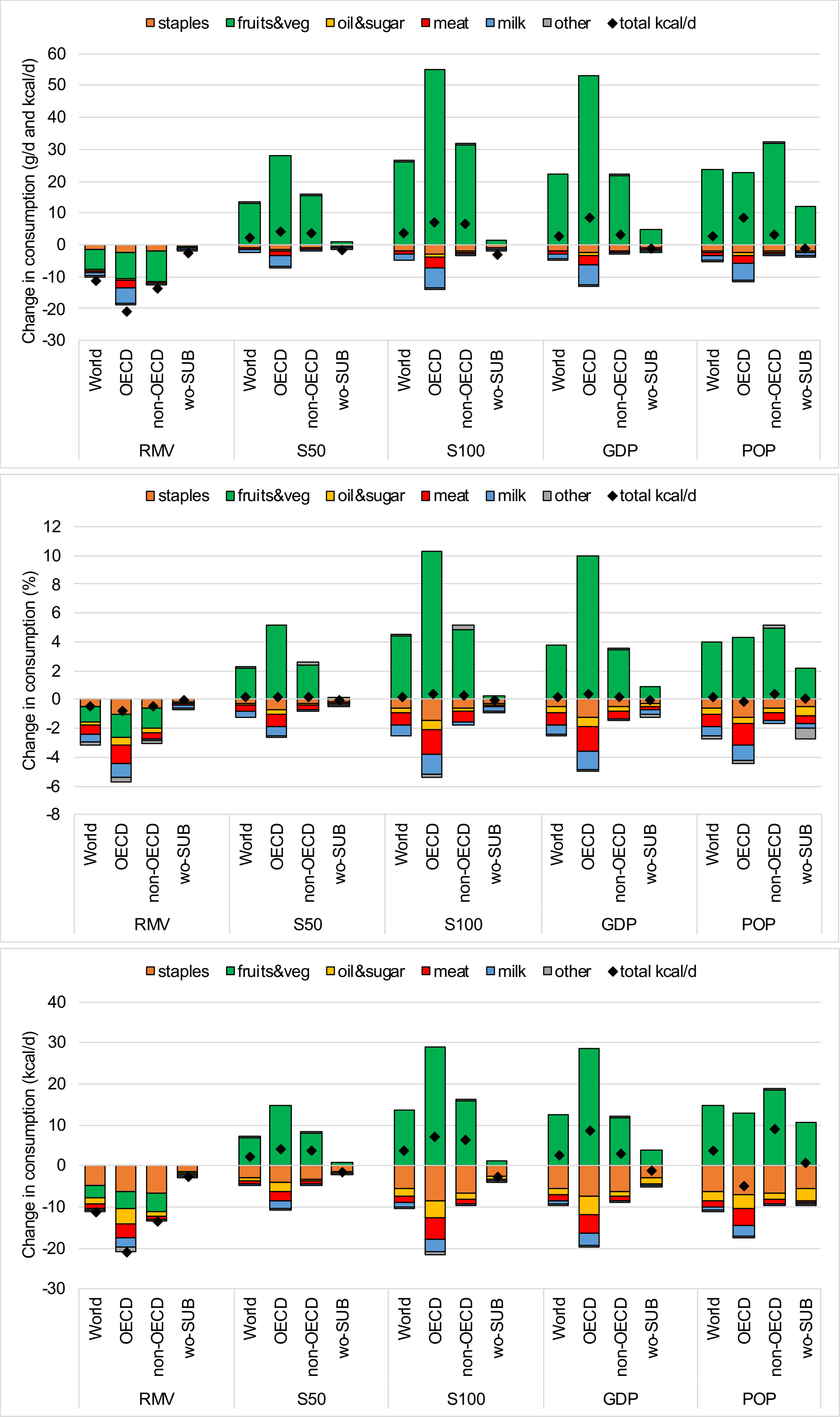

Supplementary Figure 7.

Regional changes in the consumption of horticultural products (%) by scenario.

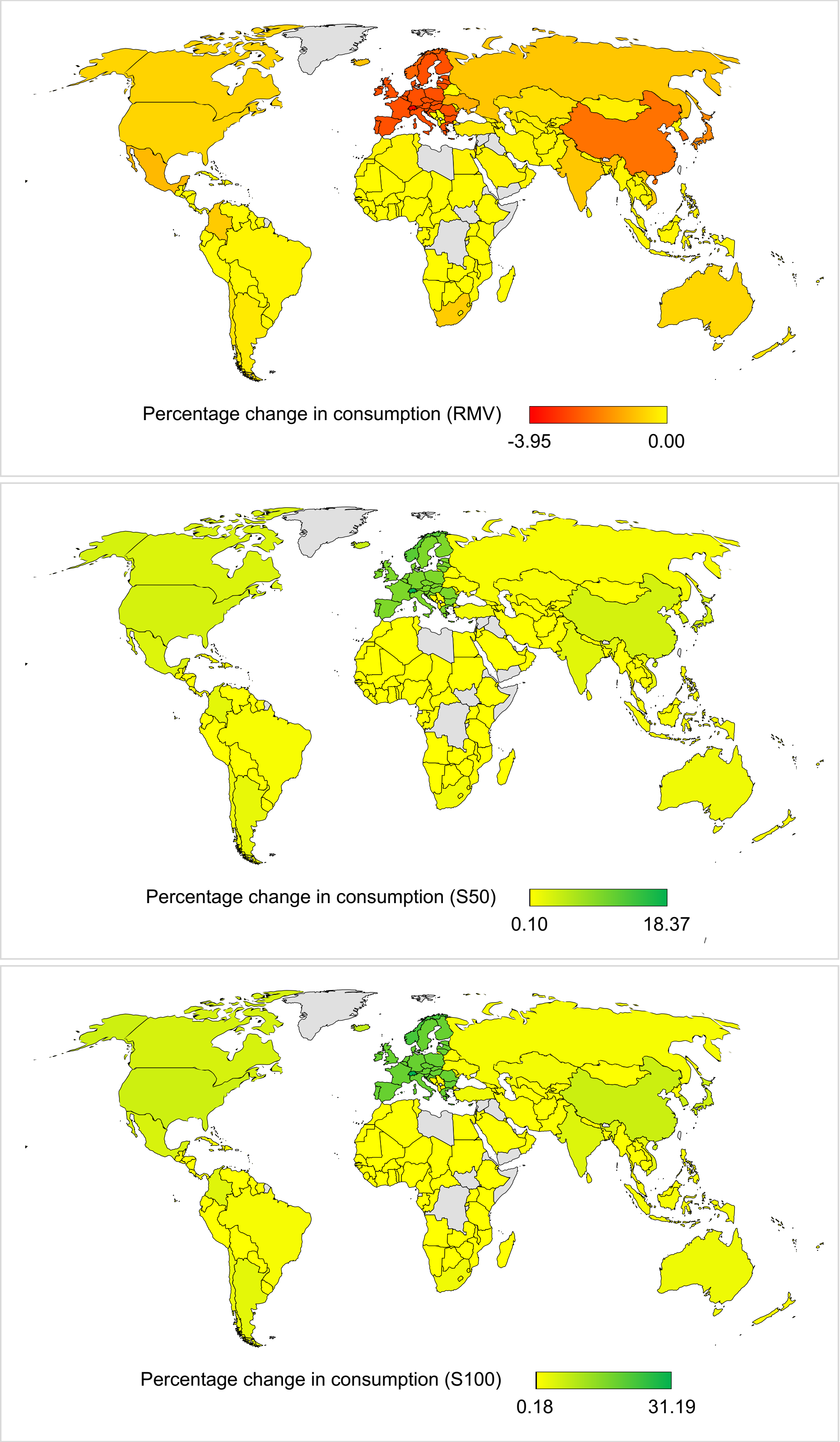

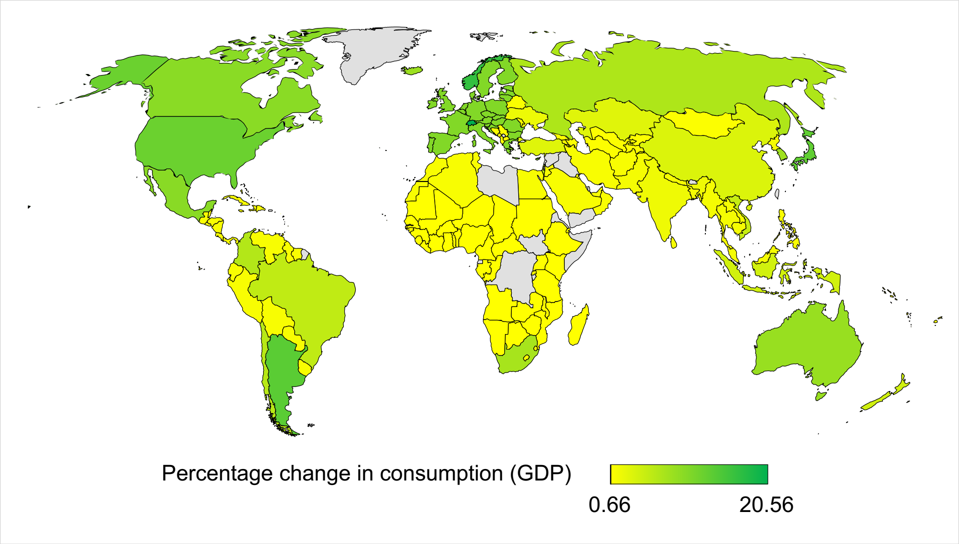
Percentage change in consumption (GDP)
0.66
20.56

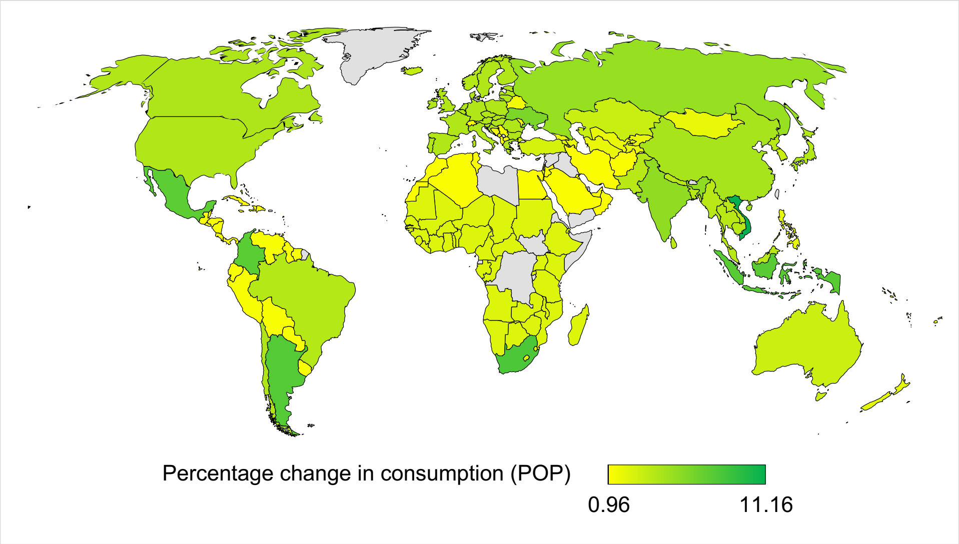
Percentage change in consumption (POP)
0.96
11.16

Supplementary Figure 8.

Absolute and percentage changes in avoidable deaths by risk factor and region in the different reform scenarios.

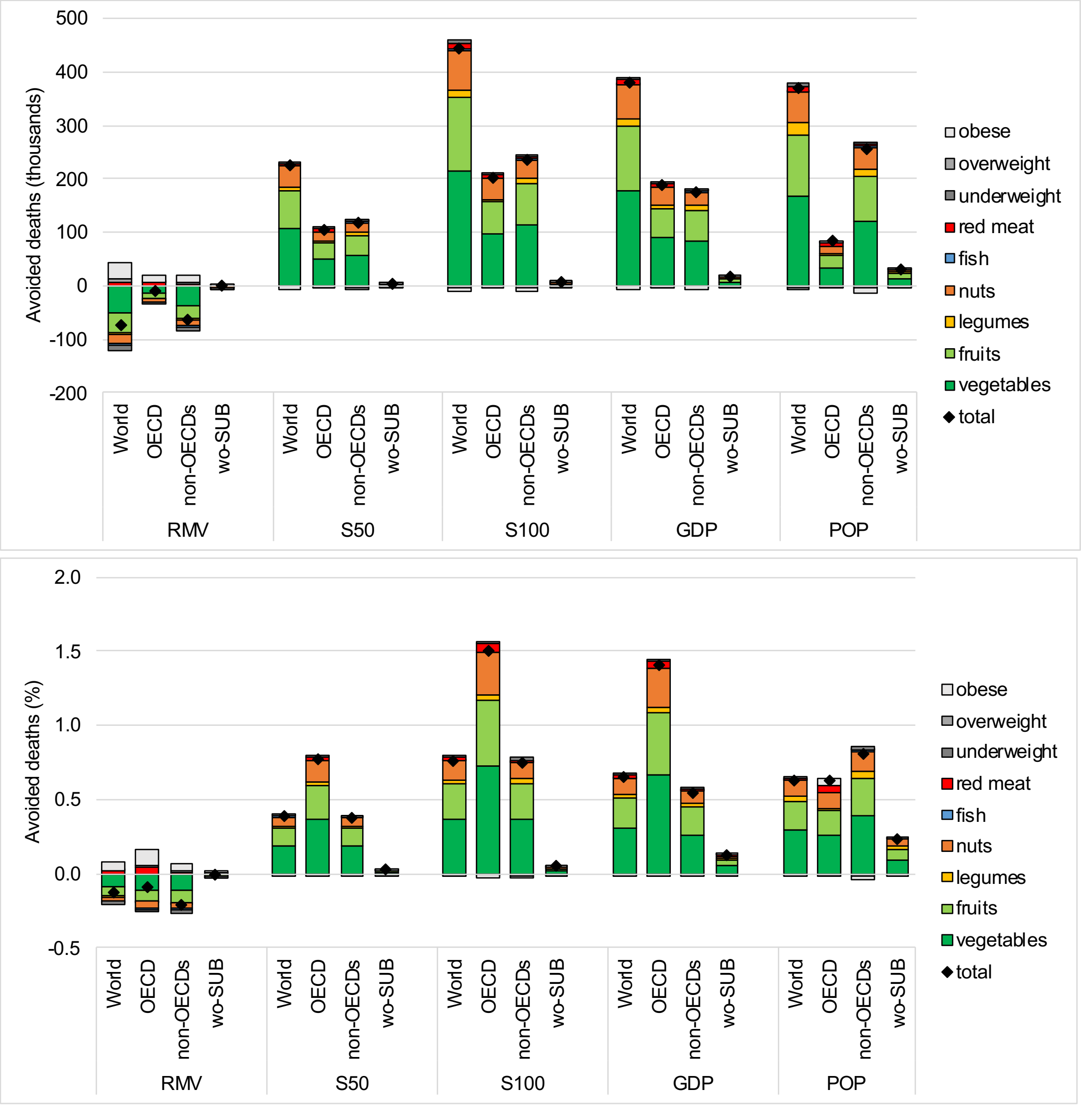

Supplementary Figure 9.

Regional changes in avoidable deaths (%) by scenario.

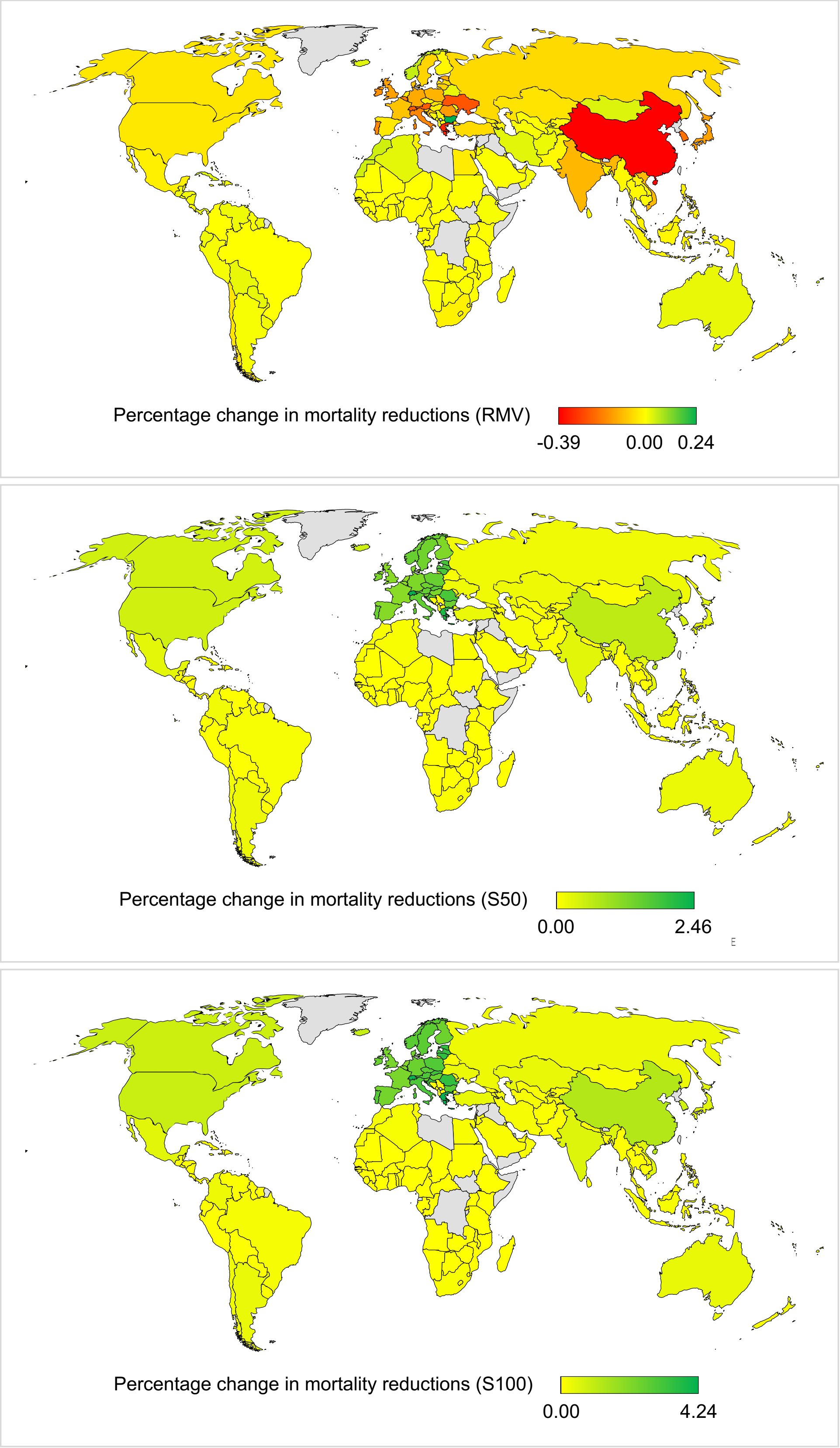

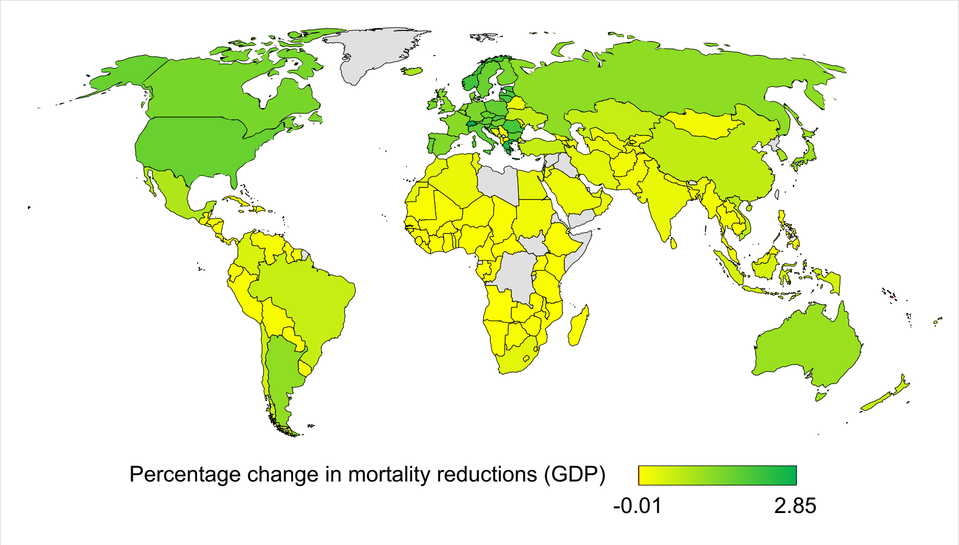
Percentage change in mortality reductions (GDP)
-0.01
2.85

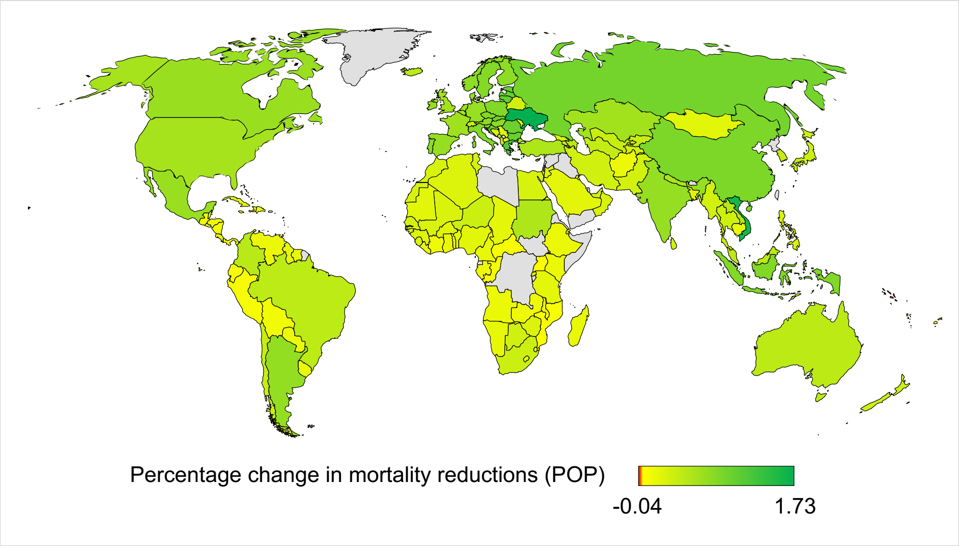
Percentage change in mortality reductions (POP)
-0.04
1.73

Supplementary Figure 10.

Absolute changes in economic welfare (measured as changes in equivalent variation) by component and region in the different reform scenarios.

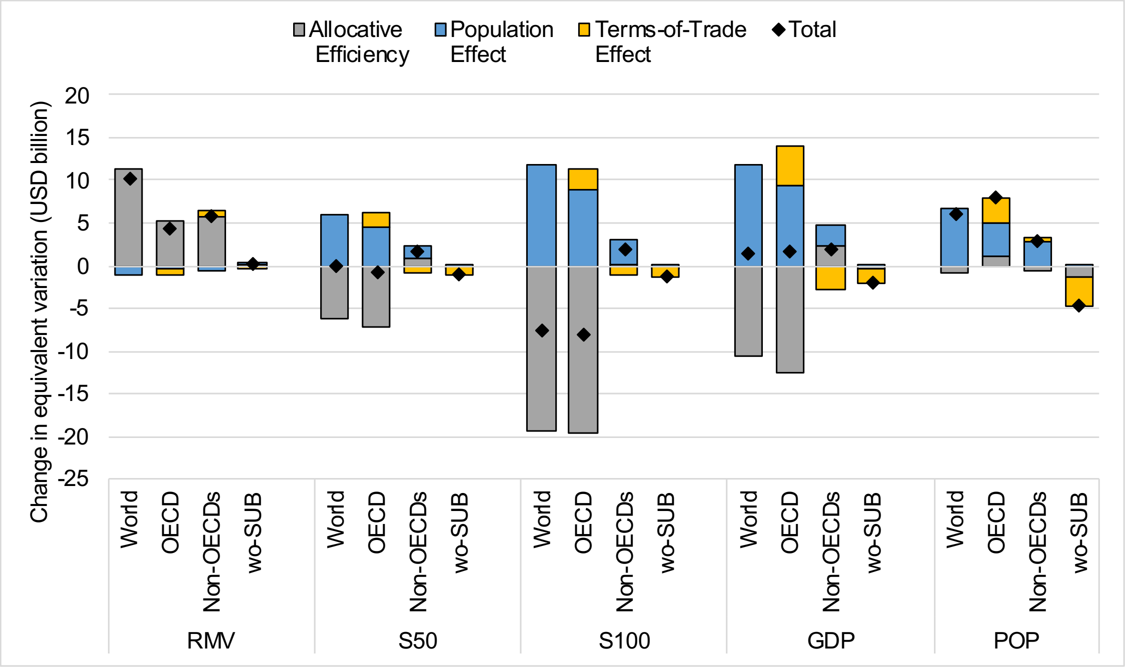

Supplementary Table 13.

Percentage changes in food production for different socio-economic development scenarios (SSPs).

Food group/ economic scenario	RMV				S50				S100				GDP				POP			
	World	OECD	non-OECD	wo-SUB	World	OECD	non-OECD	wo-SUB	World	OECD	non-OECD	wo-SUB	World	OECD	non-OECD	wo-SUB	World	OECD	non-OECD	wo-SUB
total																				
SSP2	-0.62	-1.96	-0.48	0.59	0.47	0.99	0.57	-0.33	0.93	1.89	1.10	-0.55	0.55	0.86	0.55	0.20	0.81	-1.03	1.10	2.21
SSP1	-0.52	-1.58	-0.43	0.46	0.47	1.00	0.55	-0.29	1.03	2.31	1.13	-0.61	0.66	1.27	0.59	0.13	0.93	-0.60	1.19	2.05
SSP3	-0.58	-1.94	-0.43	0.54	0.47	1.04	0.54	-0.28	0.95	2.01	1.07	-0.47	0.57	0.95	0.54	0.25	0.85	-0.97	1.11	2.23
staples																				
SSP2	-0.43	-1.64	-0.10	1.00	-0.30	-1.25	0.03	0.66	-0.58	-2.34	-0.04	1.35	-0.55	-2.20	-0.03	1.22	-0.57	-1.78	-0.22	0.80
SSP1	-0.44	-1.65	-0.11	0.97	-0.30	-1.24	0.02	0.66	-0.58	-2.33	-0.04	1.35	-0.53	-2.15	-0.06	1.28	-0.49	-1.73	-0.21	1.08
SSP3	-0.41	-1.62	-0.07	0.88	-0.30	-1.30	0.05	0.62	-0.58	-2.47	0.03	1.26	-0.54	-2.27	0.00	1.19	-0.50	-1.71	-0.20	0.89
fruits&veg																				
SSP2	-0.96	-2.83	-1.23	0.40	1.98	9.82	1.66	-1.29	3.94	18.94	3.37	-2.38	2.77	13.59	1.68	-0.68	3.61	2.39	3.73	3.99
SSP1	-0.92	-2.76	-1.16	0.34	1.98	9.80	1.66	-1.21	3.94	18.86	3.36	-2.22	2.78	13.38	1.72	-0.57	3.55	2.40	3.79	3.71
SSP3	-0.92	-2.89	-1.10	0.32	2.00	10.27	1.61	-1.22	4.01	19.94	3.27	-2.26	2.84	14.29	1.66	-0.61	3.66	2.35	3.81	4.02
oil&sugar																				
SSP2	-0.11	-1.14	0.12	0.28	-0.06	-0.79	0.12	0.15	-0.10	-1.48	0.21	0.35	-0.17	-1.80	0.20	0.37	-0.13	-1.16	0.07	0.32
SSP1	-0.09	-1.00	0.11	0.26	-0.04	-0.71	0.12	0.14	-0.07	-1.29	0.21	0.32	-0.13	-1.56	0.19	0.34	-0.09	-0.95	0.08	0.31
SSP3	-0.07	-0.98	0.13	0.25	-0.04	-0.73	0.13	0.14	-0.06	-1.36	0.24	0.32	-0.14	-1.71	0.22	0.34	-0.08	-0.94	0.09	0.29
meat																				
SSP2	-0.39	-1.51	-0.00	0.74	-0.24	-1.01	-0.00	0.60	-0.51	-2.04	-0.05	1.21	-0.49	-1.98	0.02	1.02	-0.54	-1.63	-0.13	0.47
SSP1	-0.39	-1.49	-0.00	0.70	-0.24	-1.00	0.00	0.59	-0.50	-2.01	-0.04	1.19	-0.47	-1.96	0.03	1.07	-0.48	-1.61	-0.12	0.76
SSP3	-0.38	-1.58	0.01	0.70	-0.24	-1.07	0.00	0.60	-0.50	-2.16	-0.04	1.21	-0.48	-2.10	0.01	1.08	-0.50	-1.71	-0.14	0.67
milk																				
SSP2	-0.73	-1.24	-0.62	0.24	-0.55	-0.85	-0.55	0.18	-1.09	-1.68	-1.13	0.35	-0.93	-1.58	-0.80	0.32	-0.98	-1.36	-1.15	0.23
SSP1	-0.73	-1.23	-0.64	0.24	-0.54	-0.84	-0.57	0.19	-1.09	-1.66	-1.16	0.37	-0.93	-1.56	-0.83	0.35	-0.99	-1.36	-1.19	0.26
SSP3	-0.72	-1.26	-0.64	0.24	-0.55	-0.88	-0.57	0.17	-1.10	-1.74	-1.16	0.34	-0.94	-1.63	-0.84	0.30	-1.00	-1.38	-1.20	0.17
other																				
SSP2	-1.58	-4.94	-0.81	1.89	-0.82	-3.00	-0.19	1.14	-1.79	-6.22	-0.59	2.40	-1.72	-5.90	-0.60	2.27	-1.93	-5.40	-1.34	2.09
SSP1	-0.07	-0.03	-0.14	0.01	-0.78	-2.81	-0.27	1.10	0.07	-0.09	0.29	-0.14	0.03	-0.06	0.15	-0.07	0.07	-0.10	0.22	0.03
SSP3	-1.47	-4.96	-0.92	2.06	-0.74	-3.11	-0.19	1.32	-1.62	-6.42	-0.64	2.76	-1.55	-6.04	-0.65	2.58	-1.72	-5.31	-1.31	2.20

Supplementary Table 14.

Percentage changes in food consumption for different socio-economic development scenarios (SSPs).

Food group/ economic scenario	RMV				S50				S100				GDP				POP			
	World	OECD	non-OECD	wo-SUB	World	OECD	non-OECD	wo-SUB	World	OECD	non-OECD	wo-SUB	World	OECD	non-OECD	wo-SUB	World	OECD	non-OECD	wo-SUB
total kcal/d																				
SSP2	-0.47	-0.87	-0.55	-0.11	0.09	0.17	0.15	-0.06	0.16	0.31	0.27	-0.12	0.12	0.37	0.13	-0.04	0.16	-0.20	0.37	0.03
SSP1	-0.47	-0.85	-0.54	-0.14	0.09	0.18	0.16	-0.07	0.16	0.31	0.29	-0.13	0.14	0.37	0.14	0.01	0.26	-0.20	0.38	0.34
SSP3	-0.45	-0.86	-0.50	-0.13	0.09	0.19	0.16	-0.07	0.16	0.34	0.28	-0.14	0.15	0.41	0.16	-0.01	0.25	-0.20	0.36	0.31
staples																				
SSP2	-0.48	-1.07	-0.57	-0.13	-0.28	-0.72	-0.27	-0.13	-0.58	-1.46	-0.59	-0.26	-0.55	-1.26	-0.54	-0.30	-0.63	-1.20	-0.58	-0.52
SSP1	-0.48	-1.04	-0.57	-0.15	-0.27	-0.70	-0.27	-0.13	-0.57	-1.42	-0.57	-0.26	-0.51	-1.22	-0.53	-0.22	-0.50	-1.18	-0.58	-0.16
SSP3	-0.44	-0.99	-0.50	-0.15	-0.27	-0.69	-0.26	-0.14	-0.55	-1.41	-0.54	-0.28	-0.50	-1.21	-0.49	-0.27	-0.52	-1.14	-0.54	-0.29
fruits&veg																				
SSP2	-1.09	-1.59	-1.48	-0.11	2.22	5.19	2.43	0.15	4.41	10.25	4.87	0.29	3.75	9.94	3.40	0.92	4.03	4.26	4.97	2.21
SSP1	-1.04	-1.55	-1.42	-0.12	2.19	5.21	2.45	0.11	4.34	10.24	4.91	0.20	3.70	9.86	3.43	0.82	4.00	4.27	5.01	2.13
SSP3	-1.04	-1.64	-1.38	-0.14	2.22	5.40	2.47	0.14	4.43	10.69	4.95	0.26	3.83	10.36	3.52	0.94	4.10	4.31	4.99	2.44
oil&sugar																				
SSP2	-0.25	-0.47	-0.24	-0.06	-0.15	-0.30	-0.12	-0.07	-0.34	-0.63	-0.28	-0.15	-0.35	-0.63	-0.24	-0.25	-0.44	-0.52	-0.30	-0.60
SSP1	-0.26	-0.46	-0.24	-0.10	-0.15	-0.30	-0.12	-0.07	-0.33	-0.62	-0.28	-0.14	-0.31	-0.62	-0.24	-0.14	-0.30	-0.52	-0.30	-0.10
SSP3	-0.25	-0.47	-0.22	-0.09	-0.16	-0.31	-0.12	-0.08	-0.34	-0.65	-0.28	-0.15	-0.34	-0.66	-0.25	-0.19	-0.35	-0.54	-0.32	-0.23
meat																				
SSP2	-0.64	-1.28	-0.44	-0.08	-0.44	-0.86	-0.31	-0.03	-0.90	-1.74	-0.66	-0.07	-0.85	-1.68	-0.57	-0.17	-0.85	-1.43	-0.61	-0.50
SSP1	-0.64	-1.27	-0.43	-0.12	-0.43	-0.86	-0.31	-0.03	-0.88	-1.73	-0.64	-0.07	-0.83	-1.66	-0.56	-0.09	-0.78	-1.43	-0.60	-0.14
SSP3	-0.63	-1.30	-0.42	-0.11	-0.44	-0.88	-0.32	-0.04	-0.90	-1.79	-0.66	-0.08	-0.85	-1.72	-0.57	-0.13	-0.81	-1.46	-0.62	-0.26
milk																				
SSP2	-0.45	-0.96	-0.07	-0.24	-0.34	-0.68	-0.12	-0.16	-0.67	-1.34	-0.24	-0.32	-0.62	-1.28	-0.16	-0.33	-0.59	-1.08	-0.24	-0.37
SSP1	-0.46	-0.95	-0.07	-0.27	-0.35	-0.67	-0.12	-0.18	-0.68	-1.33	-0.24	-0.35	-0.62	-1.27	-0.16	-0.32	-0.57	-1.08	-0.25	-0.25
SSP3	-0.43	-0.98	-0.05	-0.23	-0.35	-0.71	-0.12	-0.16	-0.69	-1.41	-0.24	-0.32	-0.63	-1.35	-0.16	-0.31	-0.57	-1.12	-0.26	-0.24
other																				
SSP2	-0.25	-0.40	-0.26	-0.04	0.03	-0.10	0.17	-0.03	0.03	-0.25	0.29	-0.07	-0.04	-0.18	0.16	-0.21	-0.18	-0.26	0.23	-0.75
SSP1	-0.26	-0.39	-0.27	-0.10	0.03	-0.10	0.18	-0.03	0.04	-0.25	0.32	-0.07	0.01	-0.17	0.18	-0.04	0.04	-0.25	0.25	0.04
SSP3	-0.26	-0.40	-0.26	-0.10	0.03	-0.10	0.18	-0.05	0.03	-0.26	0.32	-0.10	0.00	-0.18	0.19	-0.10	0.00	-0.26	0.25	-0.11

Supplementary Table 15.

Percentage changes in GHG emissions for different socio-economic development scenarios (SSPs).

Food group/ economic scenario	RMV				S50				S100				GDP				POP			
	World	OECD	non-OECD	wo-SUB	World	OECD	non-OECD	wo-SUB	World	OECD	non-OECD	wo-SUB	World	OECD	non-OECD	wo-SUB	World	OECD	non-OECD	wo-SUB
**total**																				
SSP2	-0.30	-1.77	-0.11	0.46	-0.17	-0.86	-0.10	0.23	-0.34	-1.72	-0.23	0.47	-0.28	-1.78	-0.02	0.41	-0.41	-1.76	-0.09	0.09
SSP1	-0.30	-1.74	-0.10	0.39	-0.16	-0.85	-0.10	0.22	-0.32	-1.69	-0.23	0.46	-0.22	-1.75	-0.00	0.49	-0.24	-1.73	-0.08	0.54
SSP3	-0.27	-1.74	-0.10	0.40	-0.16	-0.88	-0.11	0.22	-0.32	-1.75	-0.24	0.46	-0.24	-1.83	-0.03	0.48	-0.29	-1.73	-0.12	0.40
**staples**																				
SSP2	-0.45	-1.93	-0.35	0.88	-0.32	-1.57	-0.15	0.57	-0.62	-2.92	-0.37	1.18	-0.57	-2.63	-0.35	1.05	-0.58	-2.13	-0.44	0.68
SSP1	-0.46	-1.93	-0.37	0.89	-0.32	-1.55	-0.16	0.59	-0.62	-2.89	-0.38	1.22	-0.56	-2.57	-0.37	1.11	-0.52	-2.05	-0.44	0.90
SSP3	-0.40	-1.88	-0.31	0.77	-0.31	-1.64	-0.14	0.52	-0.59	-3.07	-0.32	1.07	-0.52	-2.69	-0.30	0.97	-0.49	-2.04	-0.40	0.71
**fruits&veg**																				
SSP2	-0.71	-3.51	-0.71	0.49	1.70	12.48	1.04	-1.51	3.36	23.95	2.11	-2.79	3.15	16.77	2.19	-0.64	4.44	2.52	4.96	4.18
SSP1	-0.69	-3.43	-0.68	0.44	1.70	12.43	1.03	-1.47	3.37	23.79	2.11	-2.71	3.15	16.51	2.21	-0.58	4.42	2.53	5.02	4.00
SSP3	-0.69	-3.60	-0.65	0.42	1.72	13.13	1.00	-1.48	3.42	25.37	2.05	-2.75	3.19	17.85	2.14	-0.66	4.47	2.53	5.00	4.18
**oil&sugar**																				
SSP2	-1.09	-3.56	-0.68	0.96	-0.65	-2.44	0.01	0.45	-1.39	-4.69	-0.55	1.06	-1.31	-4.83	-0.25	1.14	-1.02	-3.76	-0.63	1.32
SSP1	-1.05	-3.27	-0.70	0.88	-0.63	-2.27	-0.00	0.42	-1.32	-4.32	-0.57	0.98	-1.24	-4.40	-0.29	1.05	-0.94	-3.37	-0.62	1.24
SSP3	-1.06	-3.29	-0.75	0.94	-0.66	-2.39	-0.02	0.46	-1.38	-4.59	-0.56	1.05	-1.29	-4.72	-0.26	1.14	-0.96	-3.41	-0.69	1.28
**meat**																				
SSP2	-0.16	-1.67	0.12	0.41	-0.17	-1.03	-0.09	0.26	-0.35	-2.10	-0.20	0.52	-0.29	-2.06	0.05	0.35	-0.55	-1.78	-0.18	-0.27
SSP1	-0.17	-1.65	0.15	0.32	-0.16	-1.02	-0.08	0.24	-0.34	-2.08	-0.18	0.49	-0.23	-2.04	0.09	0.46	-0.31	-1.77	-0.16	0.35
SSP3	-0.15	-1.67	0.14	0.35	-0.16	-1.04	-0.09	0.25	-0.33	-2.13	-0.19	0.51	-0.24	-2.13	0.06	0.46	-0.37	-1.78	-0.19	0.21
**milk**																				
SSP2	-0.53	-1.33	-0.64	0.29	-0.43	-0.92	-0.58	0.20	-0.86	-1.81	-1.18	0.40	-0.69	-1.67	-0.83	0.36	-0.84	-1.46	-1.19	0.21
SSP1	-0.51	-1.31	-0.66	0.31	-0.41	-0.91	-0.59	0.22	-0.83	-1.79	-1.21	0.43	-0.65	-1.65	-0.85	0.40	-0.79	-1.45	-1.23	0.30
SSP3	-0.47	-1.29	-0.65	0.30	-0.39	-0.91	-0.60	0.20	-0.79	-1.79	-1.22	0.40	-0.63	-1.65	-0.86	0.34	-0.80	-1.42	-1.24	0.14
**other**																				
SSP2	-0.29	-0.08	-0.41	-0.03	0.22	-0.20	0.43	-0.23	0.41	-0.39	0.80	-0.44	0.16	-0.35	0.34	-0.18	0.34	-0.55	0.54	0.13
SSP1	-0.30	-0.07	-0.41	-0.02	0.24	-0.21	0.46	-0.24	0.44	-0.40	0.85	-0.45	0.18	-0.36	0.37	-0.16	0.39	-0.56	0.58	0.22
SSP3	-0.29	-0.09	-0.40	-0.01	0.24	-0.22	0.45	-0.23	0.45	-0.43	0.85	-0.44	0.19	-0.39	0.38	-0.17	0.38	-0.58	0.58	0.18

Supplementary Table 16.

Percentage changes in mortality reductions for different socio-economic development scenarios (SSPs).

Risk factor/ economic scenario	RMV				S50				S100				GDP				POP			
	World	OECD	non-OECD	wo-SUB	World	OECD	non-OECD	wo-SUB	World	OECD	non-OECD	wo-SUB	World	OECD	non-OECD	wo-SUB	World	OECD	non-OECD	wo-SUB
**all-rf**																				
SSP2	-0.13	-0.09	-0.20	0.00	0.39	0.77	0.38	0.03	0.76	1.50	0.75	0.06	0.65	1.41	0.55	0.13	0.64	0.63	0.81	0.23
SSP1	-0.12	-0.08	-0.19	0.00	0.37	0.69	0.37	0.02	0.72	1.35	0.73	0.05	0.60	1.26	0.52	0.11	0.60	0.57	0.78	0.20
SSP3	-0.13	-0.10	-0.20	0.00	0.42	0.85	0.40	0.03	0.82	1.66	0.80	0.06	0.70	1.56	0.59	0.13	0.66	0.67	0.84	0.21
**diet**																				
SSP2	-0.18	-0.19	-0.24	-0.01	0.39	0.78	0.38	0.02	0.77	1.53	0.76	0.04	0.66	1.42	0.57	0.12	0.64	0.59	0.83	0.23
SSP1	-0.16	-0.17	-0.22	-0.01	0.37	0.71	0.38	0.02	0.73	1.37	0.75	0.03	0.61	1.27	0.54	0.11	0.61	0.53	0.80	0.22
SSP3	-0.18	-0.21	-0.23	-0.02	0.42	0.87	0.41	0.02	0.83	1.70	0.81	0.05	0.71	1.58	0.61	0.12	0.67	0.63	0.86	0.24
**weight**																				
SSP2	0.05	0.11	0.03	0.02	-0.01	-0.02	-0.01	0.01	-0.01	-0.03	-0.01	0.02	-0.01	-0.01	-0.02	0.00	-0.00	0.04	-0.02	-0.00
SSP1	0.04	0.10	0.03	0.02	-0.01	-0.02	-0.01	0.01	-0.01	-0.03	-0.02	0.02	-0.01	-0.01	-0.02	0.00	-0.01	0.04	-0.02	-0.02
SSP3	0.05	0.11	0.03	0.02	-0.01	-0.02	-0.01	0.01	-0.01	-0.04	-0.01	0.02	-0.01	-0.02	-0.02	0.00	-0.01	0.04	-0.02	-0.03
**vegetables**																				
SSP2	-0.09	-0.11	-0.12	-0.01	0.19	0.37	0.18	0.01	0.37	0.72	0.36	0.02	0.31	0.67	0.26	0.05	0.29	0.26	0.39	0.09
SSP1	-0.08	-0.10	-0.11	-0.01	0.18	0.34	0.18	0.01	0.35	0.66	0.35	0.01	0.29	0.61	0.25	0.05	0.28	0.24	0.37	0.09
SSP3	-0.09	-0.12	-0.11	-0.01	0.19	0.41	0.19	0.01	0.39	0.80	0.37	0.02	0.33	0.74	0.27	0.05	0.30	0.28	0.39	0.10
**fruits**																				
SSP2	-0.06	-0.07	-0.08	-0.00	0.12	0.23	0.12	0.01	0.24	0.45	0.24	0.01	0.21	0.41	0.19	0.04	0.20	0.17	0.26	0.07
SSP1	-0.06	-0.06	-0.07	-0.00	0.12	0.21	0.12	0.01	0.23	0.41	0.24	0.01	0.20	0.38	0.18	0.03	0.19	0.16	0.26	0.07
SSP3	-0.06	-0.08	-0.08	-0.00	0.13	0.25	0.13	0.01	0.26	0.49	0.27	0.01	0.22	0.45	0.21	0.04	0.21	0.17	0.28	0.07

Supplementary Table 17.

Absolute changes in economic welfare (measured in equivalent variation, USD billion) for different socio-economic development scenarios (SSPs).

Food group/ economic scenario	RMV				S50				S100				GDP				POP			
	World	OECD	non-OECD	wo-SUB	World	OECD	non-OECD	wo-SUB	World	OECD	non-OECD	wo-SUB	World	OECD	non-OECD	wo-SUB	World	OECD	non-OECD	wo-SUB
**Total**																				
SSP2	10.2	4.2	5.8	0.1	-0.2	-0.8	1.5	-1.0	-7.6	-8.2	1.9	-1.3	1.4	1.5	1.9	-2.1	6.1	8.0	2.8	-4.7
SSP1	10.2	4.3	5.9	0.2	-1.5	-2.0	1.4	-0.9	-10.2	-10.3	1.3	-1.2	-1.1	-0.7	1.5	-1.9	3.5	6.1	1.6	-4.2
SSP3	10.8	6.5	4.7	-0.4	0.0	-0.5	1.6	-1.1	-7.6	-7.8	1.8	-1.6	1.9	2.2	1.6	-1.9	6.8	8.6	2.1	-3.9
**Allocative Efficiency**																				
SSP2	11.2	5.4	5.7	0.2	-6.2	-7.1	1.0	-0.1	-19.4	-19.6	0.2	-0.0	-10.5	-12.4	2.2	-0.3	-0.7	1.1	-0.5	-1.4
SSP1	11.1	5.3	5.8	0.1	-6.3	-7.1	0.8	-0.1	-19.7	-19.4	-0.2	-0.0	-10.4	-11.9	2.0	-0.4	-2.0	1.0	-1.1	-1.9
SSP3	11.6	6.5	5.0	0.1	-7.0	-7.7	0.7	-0.1	-21.5	-21.0	-0.4	-0.0	-12.0	-13.3	1.5	-0.2	-0.7	1.7	-1.4	-1.0
**Population effect**																				
SSP2	-1.1	-0.4	-0.7	0.0	6.0	4.6	1.4	0.0	11.8	9.0	2.7	0.0	11.8	9.3	2.4	0.1	6.8	3.9	2.8	0.1
SSP1	-0.9	-0.3	-0.6	0.0	4.8	3.7	1.2	0.0	9.5	7.1	2.3	0.0	9.3	7.3	2.0	0.1	5.5	3.1	2.3	0.1
SSP3	-0.8	-0.3	-0.5	0.0	7.1	5.6	1.4	0.0	13.9	11.0	2.9	0.0	13.9	11.3	2.5	0.1	7.5	4.4	3.0	0.1
**Terms-of-Trade effect**																				
SSP2	-0.0	-0.7	0.8	-0.1	0.0	1.7	-0.8	-0.9	0.0	2.3	-1.1	-1.3	0.0	4.6	-2.7	-1.9	0.0	3.0	0.5	-3.4
SSP1	0.0	-0.7	0.7	0.1	0.0	1.4	-0.6	-0.8	0.0	2.0	-0.9	-1.2	0.0	3.9	-2.4	-1.5	0.0	1.9	0.4	-2.4
SSP3	0.0	0.2	0.3	-0.5	0.0	1.5	-0.5	-1.0	0.0	2.3	-0.7	-1.6	0.0	4.3	-2.4	-1.8	0.0	2.5	0.5	-3.0

Supplementary References

1. Woltjer, G. B. *et al. The MAGNET model: Module description*. (2014).
2. Hertel, T. W. *Global Trade Analysis - Modeling and applications*. (Cambridge University Press, 1997).
3. Keeney, R. & Hertel, T. W. *GTAP-AGR: A Framework for Assessing the Implications of Multilateral Changes in Agricultural Policies*. (2005).
4. Haque, M. O. *Income Elasticity and Economic Development - Methods and Applications*. (Springer US, 2005). doi:10.1007/b105193.
5. Green, R. *et al.* The effect of rising food prices on food consumption: systematic review with meta-regression. *BMJ* **346**, f3703 (2013).
6. van Meijl, H., van Rheenen, T., Tabeau, A. & Eickhout, B. The impact of different policy environments on agricultural land use in Europe. *Agriculture, Ecosystems & Environment* **114**, 21–38 (2006).
7. Huang, H. Agricultural Domestic Support. in *Global Trade, Assistance, and Production: The GTAP 8 Data Base* (eds. G, B. N., Aguiar, A. & McDougall, R.) (Global Trade Analysis Project (GTAP), 2013).
8. Aguiar, A., Narayanan, B. & McDougall, R. An Overview of the GTAP 9 Data Base. *Journal of Global Economic Analysis* **1**, (2016).
9. Riahi, K. *et al.* The Shared Socioeconomic Pathways and their energy, land use, and greenhouse gas emissions implications: An overview. *Global Environmental Change* **42**, 153–168 (2017).
10. Gustavsson, J., Cederberg, C., Sonesson, U., Van Otterdijk, R. & Meybeck, A. *Global food losses and food waste: extent, causes and prevention*. (2011).
11. Food and Agriculture Organization of the United Nations. *Food balance sheets: a handbook*. (2001).
12. Springmann, M. *et al.* Options for keeping the food system within environmental limits. *Nature* **562**, 519–525 (2018).
13. Carlson, K. M. *et al.* Greenhouse gas emissions intensity of global croplands. *Nature Climate Change* **7**, 63–68 (2017).
14. Tubiello, F. N. *et al.* The FAOSTAT database of greenhouse gas emissions from agriculture. *Environmental Research Letters* **8**, 15009 (2013).
15. Troell, M. *et al.* Does aquaculture add resilience to the global food system? *Proceedings of the National Academy of Sciences* **111**, 13257–13263 (2014).
16. Chan, C. Y. *et al. Fish to 2050 in the ASEAN Region*. (WorldFish Center and Intl Food Policy Res Inst, 2017).
17. Rosegrant, M. W. *et al. Quantitative foresight modeling to inform the CGIAR research portfolio*. (Intl Food Policy Res Inst, 2017).
18. Beach, R. H. *et al.* Global mitigation potential and costs of reducing agricultural non-CO2 greenhouse gas emissions through 2030. *Journal of Integrative Environmental Sciences* **12**, 87–105 (2015).
19. Interagency Working on Social Cost of Carbon. Technical update on the social cost of carbon for regulatory impact analysis-under executive order 12866. (2013).

20. Smith, P. *et al. Agriculture, Forestry and Other Land Use (AFOLU). In: Climate Change 2014: Mitigation of Climate Change. Contribution of Working Group III to the Fifth Assessment Report of the Intergovernmental Panel on Climate Change [Edenhofer, O., et al (eds.)].* (Cambridge University Press, 2014).
21. Van Vuuren, D. P. *et al.* The representative concentration pathways: an overview. *Climatic Change* **109**, 5–31 (2011).
22. Murray, C. J. L., Ezzati, M., Lopez, A. D., Rodgers, A. & Vander Hoorn, S. Comparative quantification of health risks: conceptual framework and methodological issues. *Population Health Metrics* **1**, 1 (2003).
23. Murray, C. J. L. *et al.* GBD 2010: design, definitions, and metrics. *Lancet* **380**, 2063–2066 (2012).
24. GBD 2017 Causes of Death Collaborators *et al.* Global, regional, and national age-sex-specific mortality for 282 causes of death in 195 countries and territories, 1980–2017: a systematic analysis for the Global Burden of Disease Study 2017. *The Lancet* **392**, 1736–1788 (2018).
25. NCD Risk Factor Collaboration (NCD-RisC). Trends in adult body-mass index in 200 countries from 1975 to 2014: a pooled analysis of 1698 population-based measurement studies with 19·2 million participants. *The Lancet* **387**, 1377–1396 (2016).
26. Springmann, M., Godfray, H. C. J., Rayner, M. & Scarborough, P. Analysis and valuation of the health and climate change cobenefits of dietary change. *PNAS* **113**, 4146–4151 (2016).
27. Bechthold, A. *et al.* Food groups and risk of coronary heart disease, stroke and heart failure: A systematic review and dose-response meta-analysis of prospective studies. *Critical Reviews in Food Science and Nutrition* **59**, 1071–1090 (2019).
28. Afshin, A., Micha, R., Khatibzadeh, S. & Mozaffarian, D. Consumption of nuts and legumes and risk of incident ischemic heart disease, stroke, and diabetes: a systematic review and meta-analysis. *Am. J. Clin. Nutr.* **100**, 278–288 (2014).
29. Aune, D. *et al.* Nut consumption and risk of cardiovascular disease, total cancer, all-cause and cause-specific mortality: a systematic review and dose-response meta-analysis of prospective studies. *BMC medicine* **14**, 207 (2016).
30. Aune, D. *et al.* Fruit and vegetable intake and the risk of cardiovascular disease, total cancer and all-cause mortality–a systematic review and dose-response meta-analysis of prospective studies. *International Journal of Epidemiology* (2016).
31. Schwingshackl, L. *et al.* Food groups and risk of colorectal cancer. *International Journal of Cancer* **142**, 1748–1758 (2018).
32. Schwingshackl, L. *et al.* Food groups and risk of type 2 diabetes mellitus: a systematic review and meta-analysis of prospective studies. *European Journal of Epidemiology* **32**, 363–375 (2017).
33. Zheng, J. *et al.* Fish consumption and CHD mortality: an updated meta-analysis of seventeen cohort studies. *Public Health Nutrition* **15**, 725–737 (2012).
34. Global BMI Mortality Collaboration *et al.* Body-mass index and all-cause mortality: individual-participant-data meta-analysis of 239 prospective studies in four continents. *The Lancet* **388**, 776–86 (2016).

35. Singh, G. M. *et al.* The Age-Specific Quantitative Effects of Metabolic Risk Factors on Cardiovascular Diseases and Diabetes: A Pooled Analysis. *PLOS ONE* **8**, e65174 (2013).
36. GBD 2017 Risk Factor Collaborators *et al.* Global, regional, and national comparative risk assessment of 84 behavioural, environmental and occupational, and metabolic risks or clusters of risks for 195 countries and territories, 1990-2017: a systematic analysis for the Global Burden of Disease Stu. *Lancet* **392**, 1923–1994 (2018).
37. Micha, R. *et al.* Etiologic effects and optimal intakes of foods and nutrients for risk of cardiovascular diseases and diabetes: Systematic reviews and meta-analyses from the Nutrition and Chronic Diseases Expert Group (NutriCoDE). *PLOS ONE* **12**, e0175149 (2017).
38. GBD 2017 Diet Collaborators *et al.* Health effects of dietary risks in 195 countries, 1990-2017: a systematic analysis for the Global Burden of Disease Study 2017. *The Lancet* **0**, (2019).
39. Schwingshackl, L. *et al.* Perspective: NutriGrade: A Scoring System to Assess and Judge the Meta-Evidence of Randomized Controlled Trials and Cohort Studies in Nutrition Research. *Advances in Nutrition: An International Review Journal* **7**, 994–1004 (2016).
40. World Cancer Research Fund/American Institute for Cancer Research. Diet, Nutrition, Physical Activity and Cancer: A Global Perspective. Continuous Update Project Expert Report. (2018).
